# First molecular detection and complete sequence analysis of porcine circovirus type 3 (PCV3) in Peninsular Malaysia

**DOI:** 10.1371/journal.pone.0235832

**Published:** 2020-07-24

**Authors:** Chew Yee Tan, Keerati Opaskornkul, Roongroje Thanawongnuwech, Siti Suri Arshad, Latiffah Hassan, Peck Toung Ooi

**Affiliations:** 1 Department of Veterinary Clinical Studies, Faculty of Veterinary Medicine, Universiti Putra Malaysia, Serdang, Selangor, Malaysia; 2 Department of Veterinary Pathology, Faculty of Veterinary Science, Chulalongkorn University, Pathumwan, Bangkok, Thailand; 3 Department of Veterinary Pathology & Microbiology, Faculty of Veterinary Medicine, Universiti Putra Malaysia, Serdang, Selangor, Malaysia; 4 Department of Veterinary Laboratory Diagnostics, Faculty of Veterinary Medicine, Universiti Putra Malaysia, Serdang, Selangor, Malaysia; Panstwowy Instytut Weterynaryjny - Panstwowy Instytut Badawczy w Pulawach, POLAND

## Abstract

Porcine circovirus type 3 (PCV3) is a newly emerging virus in the swine industry, first reported recently in 2016. PCV3 assembles into a 2000 bp circular genome; slightly larger than PCV1 (1758–1760 bp), PCV2 (1766–1769 bp) and PCV4 (1770 bp). Apart from being associated with porcine dermatitis and nephropathy syndrome (PDNS), PCV3 has been isolated from pigs with clinical signs of reproductive failures, myocarditis, porcine respiratory disease complex (PRDC) and neurologic disease. Given that PCV3 is increasingly reported in countries including Thailand and U.S. with whom Malaysia shares trade and geographical relationship; and that PCV3 is associated with several clinical presentations that affect productivity, there is a need to study the presence and molecular characteristics of PCV3 in Malaysian swine farms. Twenty-four commercial swine farms, three abattoirs and retail shops in Peninsular Malaysia were sampled using convenience sampling method. A total of 281 samples from 141 pigs, including 49 lung archive samples were tested for PCV3 by conventional PCR. Twenty-eight lung samples from wild boar population in Peninsular Malaysia were also included. Nucleotide sequences were analyzed for maximum likelihood phylogeny relationship and pairwise distances. Results revealed that PCV3 is present in Peninsular Malaysia at a molecular prevalence of 17.02%, with inguinal lymph nodes and lungs showing the highest molecular detection rates of 81.82% and 71.43% respectively. Despite wide reports of PCV3 in healthy animals and wild boars, no positive samples were detected in clinically healthy finishers and wild boar population of this study. PCV3 strain A1 and A2 were present in Malaysia, and Malaysian PCV3 strains were found to be phylogenetically related to Spanish, U.S. and Mexico strains.

## Introduction

Circoviruses of swine comprise of porcine circovirus type 1 (PCV1), porcine circovirus type 2 (PCV2) porcine circovirus type 3 (PCV3) and most recently reported porcine circovirus type 4 (PCV4). PCV1 was discovered in 1974 as a cell culture contaminate [1]. In contrast, PCV2 is associated with a group of complex multi-factorial diseases classified under the umbrella term of Porcine circovirus associated diseases (PCVAD) [2]. The novel detection of PCV4 was described in a herd with severe clinical signs of respiratory disease, enteritis and porcine dermatitis and nephropathy syndrome (PDNS) [3].

PCV3 is a newly emerging virus in the swine industry, first reported in 2016 in the United States [4]. PCV3 assembles into a 1999–2001 bp circular genome; slightly larger than other known porcine circoviruses which ranged from 1758–1760 bp (PCV1) and 1766–1769 bp (PCV2) [5–8]. PCV4, very recently reported in Hunan province, China, was described to be 1770 bp [3]. Albeit their different lengths, genome of all porcine circoviruses encodes for three known proteins: replication-associated (Rep) protein, capsid (Cap) protein and open reading frame (ORF) 3, which function has yet to be determined. International Committee on Taxonomy of Viruses (ICTV) defines a distinct *Circovirus* species based on sequence similarity: a novel circovirus must share < 75% nucleotide (nt) identity over its entire genome and < 70% amino acid (aa) identity of its Cap protein with other species in the genus [9]. PCV1 and PCV2 shares < 80% similarity of overall nt identity, 86% and 65% similarity of aa identity in their ORF1 and ORF2 respectively [10–12]. In a complete genome of PCV3, ORF1 and ORF2 genes encode for 296 – 297aa Rep protein and 214aa Cap protein respectively [4, 7]. PCV3 shares even lower similarity of only < 50% of overall nt identity, 48% and 26–36% aa identity of ORF1 and ORF2 respectively with PCV2 [4, 9, 13]. The genome of PCV4 showed 50.3%, 51.5% and 43.2% nt similarities to PCV1, PCV2 and PCV3 respectively. Most strikingly, aa identities of Cap protein of PCV3 and PCV4 differ by 75.5% [3]. While the conserved nonanucleotide stem loop motif (T/n)A(G/t)TATTAC representing the origin of replication can be found in all porcine circoviruses, their motif differ. PCV1 and PCV3 have an identical motif of TAGTATTAC [4, 9, 14] whereas the motif on PCV4 *rep* gene was shown to be CAGTATTAC [3]. PCV2 has a nonamer sequence unique among circovirus species–AAGTATTAC [14].

PCV3 was associated with a PDNS outbreak in North Carolina, where there was an increase in sow mortality rate and decrease in conception rate, presented with skin and kidney lesions suggestive of PDNS [4]. Several other clinical presentations have been associated with PCV3, including reported reproductive failures [15], neonatal congenital tremor [16], myocarditis and multi-organ inflammation [13]. Role of PCV3 in porcine respiratory disease complex (PRDC) has also been discussed [13, 17]. Although many common swine pathogens especially PCV2, and others such as porcine reproductive and respiratory syndrome virus (PRRSV) and ungulate protoparvovirus 1 (PPV) have been ruled out in these reports, there could still be other co-infections that elude the role of PCV3 in pathogenesis. A successful reproduction of PDNS-like clinical disease following experimental inoculation of 4- and 8-week-old specific-pathogen-free piglets with infectious PCV3 DNA clone has been demonstrated, thus implying that PCV3 may have a direct role in disease process [18]. Considering the presence of PCV3 in increasing number of countries including Thailand [17, 19] and U.S. [4, 13] with whom Malaysia shares trade and geographical relationship; and that PCV3 is associated with several clinical presentations that affect productivity, molecular prevalence of PCV3 in Malaysia and molecular characteristics of Malaysian PCV3 strains reported in this study may contribute practical knowledge to the Malaysian swine farming industry.

## Materials and methods

### Sample collection

Commercial swine farms involved in this study were located in Perak, Selangor, Melaka and Johor states representing different regions of Malaysia. From these 24 farms, 123 pigs were subjected to convenience sampling method. From three abattoirs and retail shops, 18 clinically healthy finishers were sampled by random selection. A grand total of 141 pigs were included in this study. The sampled animals were also categorized by age group (foetuses, piglets, weaners, growers or finishers and sows), health status (clinically healthy or clinically ill), standing sow population of origin farm (< 800 or > 800 heads) and distance between origin farm and neighbouring farms (< 1km, 1 – 10km, > 10km). The 281 organ samples collected were comprised of 49 archived lung samples of year 2016–2017, 18 lung samples from clinically healthy finishers and 214 tissues samples of various organs from clinically ill pigs of year 2018–2019. For each animal sampled, at least lung and/or inguinal lymph node was collected. Other organs (spleen, tonsil, kidney, heart, mesenteric lymph nodes, liver and brain) were collected on the basis of availability. Clinically ill pigs were those showing various clinical signs such as wasting, moribund, dyspnea, neurological signs and sudden death. Majority of the sampled pigs were of weaner and grower stage between the age of 4–12 weeks old. Detailed breakdown of each sampled farm and pig is provided in S1 and S2 Tables. In addition, 28 archived wild boar lung samples of year 2018–2019 were also included in this study.

This study was granted approval from the Universiti Putra Malaysia (UPM) Institutional Animal Care and Use Committee (IACUC) under AUP Code UPM/IACUC/AUP-R030/2019 and was conducted adhering to the guidelines as stated in the Code of Practice for Care and use of Animals for Scientific Purposes as stipulated by Universiti Putra Malaysia. All samples were collected under the supervision of veterinarians from Faculty of Veterinary Medicine, Universiti Putra Malaysia.

### Molecular prevalence of PCV3

DNA extraction was performed using DNeasy Blood & Tissue Kit extraction kit (Qiagen, Germany) in accordance to manufacturer’s instructions. Conventional PCR was performed to amplify the ORF2 region of PCV3, by using MyTaq™ Red Mix 2X (Bioline, United Kingdom) and published primers, KF–5’–TTACTTAGAGAACGGACTTGTAAC G–3’ and KR–5’–AAATGAGACACAGAGCTATATTCAG–3’ [15]. Briefly, 12.5 μL of Taq DNA polymerase master mix and 1.0 μM each from the primer pair were used in a 25 μL total PCR reaction volume. Cycling conditions of the conventional PCR were as described by Ku *et al*. [15]. PCR products were stained using RedSafe™ nucleic acid staining solution (iNtRON Biotechnology, South Korea) and analysed by agarose electrophoresis. Expected PCR product was a 649 bp band indicating PCV3 *cap* gene sequence spanning from nt position 1343–1987. Samples that showed a positive band at the 650 bp region as marked by GelPilot 100 bp Plus Ladder (Qiagen, Germany) were further sequenced (Macrogen, South Korea).

To test for association between PCV3 molecular detection status and age group, health status, farm standing sow population and distance from neighbouring farms, Chi‐square tests were performed with statistical significance level set at p < 0.05. Fisher's exact tests were run in place of Chi‐squared tests for variables with expected count of less than five. Post hoc tests of cell residuals were ran on statistically significant chi-square and Fisher's exact test values, with p-values adjusted with Bonferroni correction. Animals that tested positive for PCV3 in lung and/or inguinal tissues, with at least one other organ samples (spleen, tonsil, kidney, heart, mesenteric lymph nodes, liver and/or brain) tested for PCV3 were identified. Molecular detection rate comparison across tissues from different organs was then similarly evaluated as described above. All statistical tests were performed using IBM SPSS Statistics for Windows v23 software programme [20].

### Nucleotide sequence and complete genome analysis

**Fig 1:**
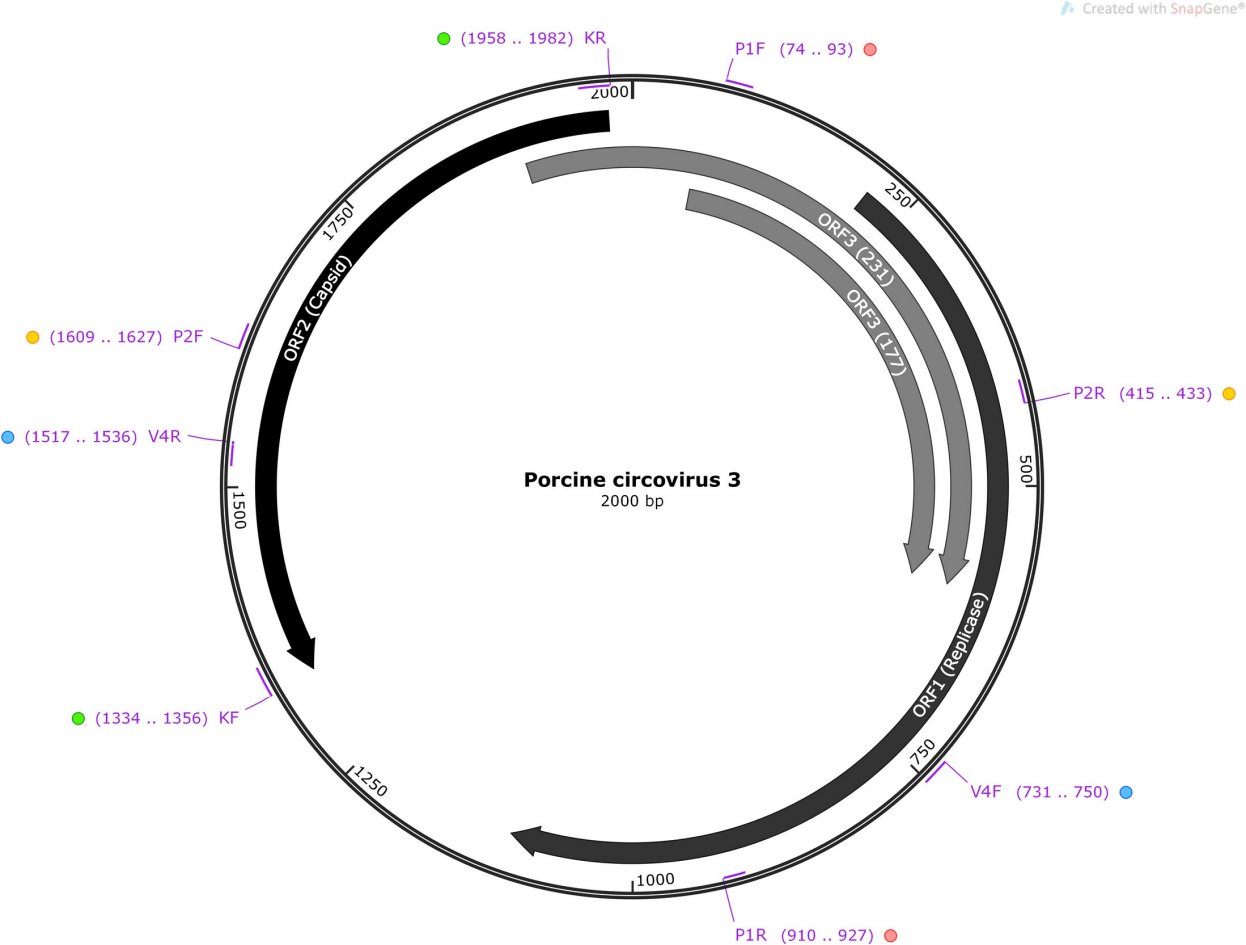
Schematic representation of primer pairs used in this study to generate complete genome sequence of PCV3.

Upon confirmation of the nt sequences as *cap* gene of PCV3 by NCBI Nucleotide BLAST^®^ [21], the positive samples were subjected to PCR with another three pairs of primers (Fig 1, Table 1) to generate the complete 2000 bp genome of PCV3. Two pairs of published sequencing primers [4] were run under modified cycling conditions, chiefly adjusting annealing temperature and final extension time as detailed in Table 1. Primer pair V4F/V4R were designed based on PCV3 strain KY075986 [15], using Primer-BLAST^®^ [22]. All PCR assays were performed using the same reagents, reaction volumes and primer concentration as described in method of PCV3 *cap* gene detection. PCR protocols used in this study is accessible at http://dx.doi.org/10.17504/protocols.io.bdd9i296 [PROTOCOL DOI].

**Table 1:** PCR cycling condition and primers used in this study to generate complete genome sequence of PCV3.

Primer Pair	Nucleotide Sequence (5’– 3’)	Product Length (bp)	PCR Cycling Condition (Temperature / Time)						Reference
			Initial Denaturation	Number of Cycle	Denaturation	Annealing	Extension	Final Extension	
P1F	CACCGTGTGAGTGGATATAC	854	94⁰C / 4 min	35	94⁰C / 20 s	55⁰C / 30 s	72⁰C / 30 s	72⁰C / 5 min	[4]
P1R	CAAACCCACCCTTAACAG								
P2F	GTCGTCTTGGAGCCAAGTG	807	95⁰C / 5 min	35	94⁰C / 30 s	62⁰C / 30 s	72⁰C / 1 min	72⁰C / 7 min	[4]
P2R	CGACCAAATCCGGGTAAGC								
KF	TTACTTAGAGAACGGACTTGTAACG	649	94⁰C / 5 min	35	94⁰C / 30 s	55⁰C / 30 s	72⁰C / 1 min	72⁰C / 10 min	[15]
KR	AAATGAGACACAGAGCTATATTCAG								
V4F	GAAAACGCGGGAAGCTTGTG	806	95⁰C / 5 min	35	94⁰C / 30 s	56⁰C / 30 s	72⁰C / 1 min	72⁰C / 10 min	Designed in this study
V4R	CCACTTCTGGCGGGAACTAC								

Sequencing was done to confirm the identity of the PCV3 nt sequences. Sequence assembly and multiple sequence alignment were generated using MEGA v7.0.26 software programme [23]. The resulting sequences were analysed using NCBI Nucleotide BLAST^®^ [21] for a final identity confirmation as PCV3 by comparing their similarity with reference PCV3 sequences deposited in the GenBank. Maximum likelihood (ML) phylogenetic trees were constructed with MEGA7 programme, using 1000 bootstrap replicates with either General Time Reversible (GTR) model for species-specific circoviruses comparison or Tamura-Nei model for porcine circoviruses analyses. Pairwise distance analysis with p-distance model was performed using the same software, similarly with 1000 bootstrap replicates. Both transitions and transversions nt substitutions were included. After number of base differences per site was computed, percentage nt identities were calculated by subtracting the computed p-distance values from 1.0 and multiplying by 100.

The same 45 PCV3 strains included in the phylogenetic methods were further analysed. Tajima’s D, Fu and Li’s D, and Fu and Li’s F statistical tests of neutrality were performed on nt sequences of PCV3 ORF1 and ORF2 genes using DnaSP v6.12.03 software programme [24–26]. DNA polymorphism data of pairwise nt differences were analysed to measure balancing selection and negative selective processes over the sequences. Statistical significance was set at p ≤ 0.05 for all three tests. Shannon's entropy H(x) values were calculated with BioEdit software v7.2.5 using the Shannon entropy formula: $-(\sum_{j=1}^{4}p_{ij}{log}_{2}p_{ij})$ where *i; j* is equal to 1, 2, 3 and 4, corresponding to A, C, G and T nt and *pij* being the proportion of nt *j* in site *i*. Entropy plots of aa sequences of the ORF1 and ORF2 genes were constructed to plot the diversity of aa residues at a given position [27, 28]. Range, mean and standard error of mean (SEM) were calculated using IBM SPSS Statistics for Windows v22 software programme. Positive and negative selective pressures acting specifically on each codon of the ORF1 and ORF2 nt sequences were estimated based on calculated difference between non-synonymous (dN) and synonymous (dS) substitution rates per codon. Single-likelihood ancestor counting (SLAC), fixed-effects likelihood (FEL), internal branches fixed-effects likelihood (IFEL), fast, unconstrained Bayesian approximation (FUBAR) and mixed effects model of evolution (MEME) selection pressure methods were run in the DataMonkey web server (http://www.datamonkey.org/) [29–32]. To infer dN and dS rates, FUBAR uses Bayesian approach; FEL, IFEL and MEME utilize ML approach; while SLAC incorporates additional counting approaches. FEL, IFEL, SLAC and FUBAR detects both positive and negative selection, but MEME aims to detect aa sites evolving under positive selection. Comparison between rates of dN and dS substitutions were expressed as dN–dS < 0, = 0 and > 0 (dN / dS < 1, = 1 and > 1 for MEME method) and interpreted as indication of negative selection, neutral evolution and positive selection respectively. Statistical significance was set at p ≤ 0.05 for FEL and IFEL methods and p ≤ 0.1 for SLAC and MEME method. FUBAR method was run with posterior probability of 0.9. The dN–dS and H(x) entropy values for every codon were plotted against their respective aa positions along the ORF1 and ORF2 genes.

## Results

### Molecular prevalence of PCV3

Out of the 141 pigs, 24 pigs from 10 farms were positive for PCV3 based on PCR detection (Table 2), representing a molecular prevalence of 17.02% of PCV3 in Peninsular Malaysia. PCR results for each 141 animals are provided in S2 Table. Notably, all 18 samples from clinically healthy pigs and all 28 lung samples from the wild boar population were tested negative for PCV3. Statistical significance was observed between PCV3 molecular detection status and age group (p: 0.022), as well as health status (p: 0.043) of the test animals. In the age group set of variables, only the weaners group (p: 0.0007) was shown to be statistically significant at adjusted p < 0.005. No statistical differences in the frequency of animals molecularly positive for PCV3 were observed across farms with different standing sow population (p: 0.180) and distance from neighbouring farms (exact p: 0.512). Statistical significance for all Chi-square and Fisher’s exact tests is summarized in Table 3. Detailed results of statistical tests are tabulated in S3 Table.

**Table 2:** Distribution of PCV3 positive farms and animals in Peninsular Malaysia as detected by PCR assay.

Region	State	No. of Positive Farm(s) / Tested Farms	No. of Positive Animal(s) / Tested Animals
Northern	Penang	1 / 4	1 / 16
	Perak	3 / 5	4 / 30
Central	Selangor	4 / 9	16 / 81
Southern	Melaka	1 / 3	2 / 9
	Johor	1 / 3	1 / 5
Total		10 / 24	24 / 141

**Table 3:** Statistical significance for Chi-square and Fisher’s exact tests evaluating association between PCV3 molecular detection status and age group, health status, farm standing sow population, distance from neighbouring farms, and across different organs. Statistically significant values are highlighted with grey boxes and bold typeface.

Comparison Variable	Standing Sow Population	Neighbouring Farm Proximity Radius	Clinical Health Status	Age Group					Organ								
Pearson Chi-Square (p < 0.05)	0.180	0.449	**0.040**	**0.024**					**0.049**								
Fisher's Exact Test (p < 0.05)	0.180	0.512	**0.043**	**0.022**					**0.047**								
Bonferroni correction adjusted p-value	N/A	N/A	N/A	0.005					0.003								
Cell residuals adjustment post hoc (p < adjusted)	N/A	N/A	N/A	**T**	**P**	**W**	**G**	**F**	**L**	**I**	**S**	**T**	**K**	**H**	**M**	**V**	**B**
				0.271	0.317	**0.0007**	0.134	0.036	0.029	**0.0002**	0.855	0.450	0.807	0.105	0.077	0.008	0.567

Key:

• Age group: T–fetus, P–piglet, W–weaner, G–grower, F–finisher and sow

• Tissue type: L–lung, I–inguinal lymph node, S–spleen, T–tonsil, K–kidney, H–heart, M–mesenteric lymph node, V–liver, B–brain.

• N/A: Not applicable.

The molecular detection rate across different organs (lung, inguinal lymph node, mesenteric lymph node, spleen, tonsils, kidney, heart, liver, brain) was tabulated for the positive animals (Table 4). For an accurate representation of the distribution, positive animals with only one organ sample type tested were excluded from the analysis. The presence of PCV3 genetic material was detected in all nine group of organ samples included in this study. The highest frequency of PCR positive results was observed in inguinal lymph node and lung samples, with detection rates of 81.82% and 71.43% respectively. For other organs, positive PCR detection rate ranged from 12.5% to 54.55%. PCR detection rate was moderately high in five organs, namely tonsil, spleen, kidney, mesenteric lymph node and brain. The lowest detection rates were seen in heart and liver. In terms of statistical significance, it was found that only the inguinal lymph nodes group (p: 0.0002) was statistically significant at adjusted p < 0.003, as shown in Table 3.

**Table 4:** PCV3 PCR detection rate across different organs types.

Organ	No. of Positive Sample(s) / Tested Sample	PCR Detection Rate (%)
Lung	10 / 14	71.43^a^
Inguinal lymph node	9 / 11	81.82^b^
Mesenteric lymph node	4 / 12	33.33 ^a^
Spleen	7 / 14	50.00 ^a^
Tonsil	6 / 11	54.55 ^a^
Kidney	5 / 11	45.45 ^a^
Heart	1 / 6	16.67^a^
Liver	1 / 8	12.50^a^
Brain	1 / 4	25.00^a^

^a, b^ Different superscript letters indicate significant differences (adjusted p-value < 0.003) among different organ samples.

### Nucleotide sequence and complete genome analysis

Twelve complete genome sequences and three *cap* gene sequences were obtained successfully. The local PCV3 sequences showed over 99% similarity with PCV3 sequences recorded in GenBank. The genome of Malaysian PCV3 strains in this study is 2000 bp in length, encoding a 296 aa Rep protein and a 214 aa Cap protein.

**Fig 2:**
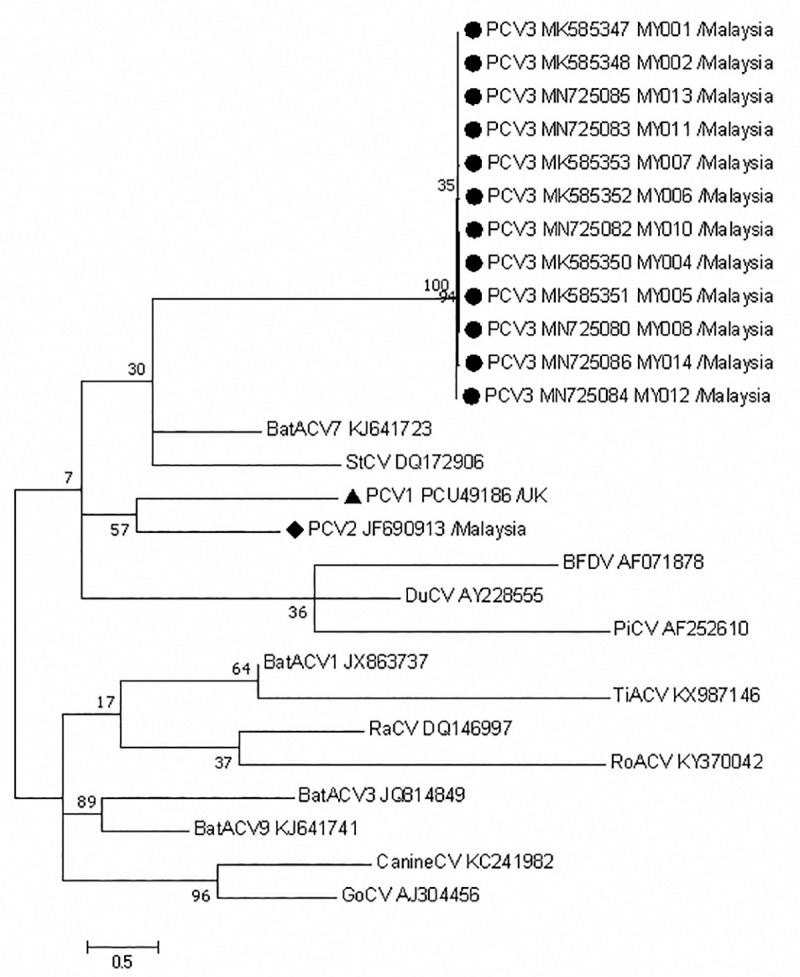
Phylogenetic analysis of complete genome sequences of PCV3 and known member species of *circovirus* genus. Twelve Malaysian PCV3 strains (●) were compared with PCV1 (▲), PCV2 (◆) and 13 circovirus member species (Bat associated circovirus, BatACV; beak and feather disease virus, BFDV; canine circovirus, CanineCV; duck circovirus; DuCV; goose circovirus, GoCV; pigeon circovirus, PiCV; raven circovirus, RaCV; rodent associated circovirus, RoACV; starling circovirus, StCV; tick associated circovirus, TiACV). GenBank accession numbers are indicated at the end of each species strain. The tree was constructed using ML method, GTR model with gamma distribution and invariant sites with tree topology evaluated with 1000 bootstrap replicates. The scale bar indicates branch length measured in number of substitutions per site.

ML phylogenetic trees were constructed to analyze Malaysian PCV3 strains in relation to known member species of *circovirus* genus using nt sequences of exemplar isolates listed by ICTV [33]. PCV1 and PCV2, as well as PCV3 strains from different countries were also included in the analysis. The phylogenetic relationship among species in *Circoviridae* was inferred using ML method based on GTR model (Fig 2). PCV1, PCV2 and PCV3 were clustered into a same clade with >50% bootstrap value. Supported by a lower bootstrap value of 30%, Malaysian strains of PCV3 were phylogenetically related to bat associated circovirus 7 (BatACV7) and starling circovirus (StCV).

**Fig 3:**
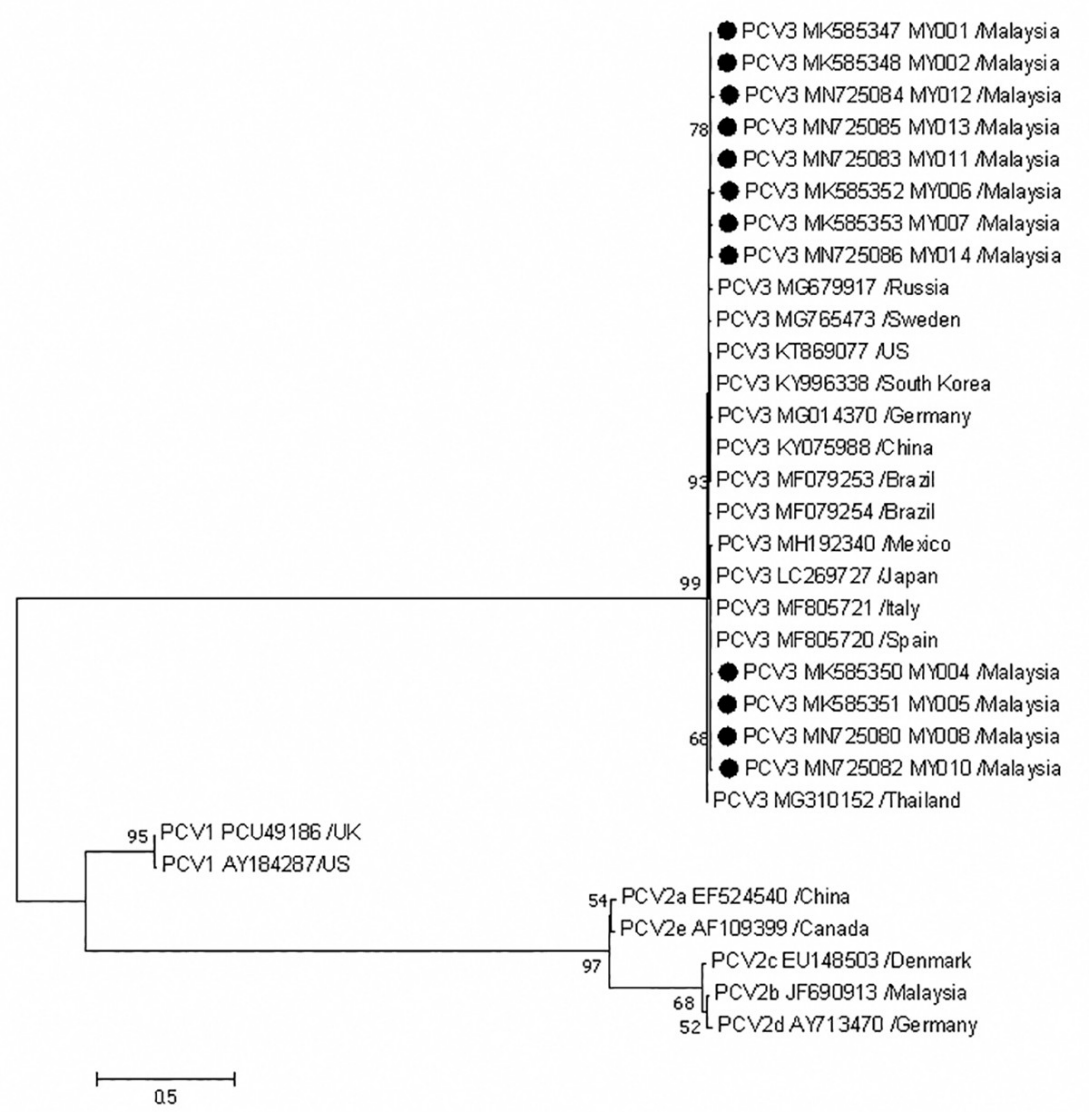
Phylogenetic analysis of complete genome sequences of PCV1, PCV2 and PCV3. Twelve Malaysian PCV3 strains (●) were compared with 5 PCV2 strains and 2 PCV1 strains. GenBank accession numbers and origin country are as indicated. Malaysian PCV3 strains were additionally labelled with strain ID. The tree was constructed using ML method, Tamura-Nei model evaluated with 1000 bootstrap replicates. The scale bar indicates branch length measured in number of substitutions per site.

The phylogenetic relationship among porcine circoviruses was inferred using ML method based on Tamura-Nei model (Fig 3). All PCV3 strains analyzed in this study were clustered in a distinct clade with the longest branch length, separated from PCV1 and PCV2.

Further, percentage nt identities between 42 PCV3 sequences (12 Malaysian sequences and 30 GenBank reference strains) were compared using Pairwise Distance method with p-distance model (S4 and S5 Tables). All 2000 nt positions were included in the final dataset. Overall, all 42 PCV3 sequences in the comparison dataset are closely related to each other, given that only 0.58% (5 / 861) of the p-distance values are ≥ 0.020. The p-distance values range from 0.002 to 0.021, averaging at 0.010. This is equivalent to a shared percentage nt identities of 97.9%– 99.8%. Among the 12 Malaysian sequences, while the p-distance range maintains, the average is slightly higher at 0.012.

A similar pairwise distance analysis was run to compute the p-distance values among *cap* gene sequences of 15 Malaysian PCV3 strains and 30 PCV3 GenBank reference strains (S6 and S7 Tables). The range of p-distance values widens from 0.000 to 0.026, with an increased average of 0.013. This is equivalent to a shared percentage nt identities of 97.36%– 100%. Compared to the complete genomes, the *cap* gene sequences are less closely related to each other, given that 8.28% (82 / 990) of the p-distance values are ≥ 0.020. Among the 15 Malaysian sequences, the p-distance range is slighter smaller at 0.000 to 0.020, and the average is slightly lower at 0.012.

**Fig 4:**
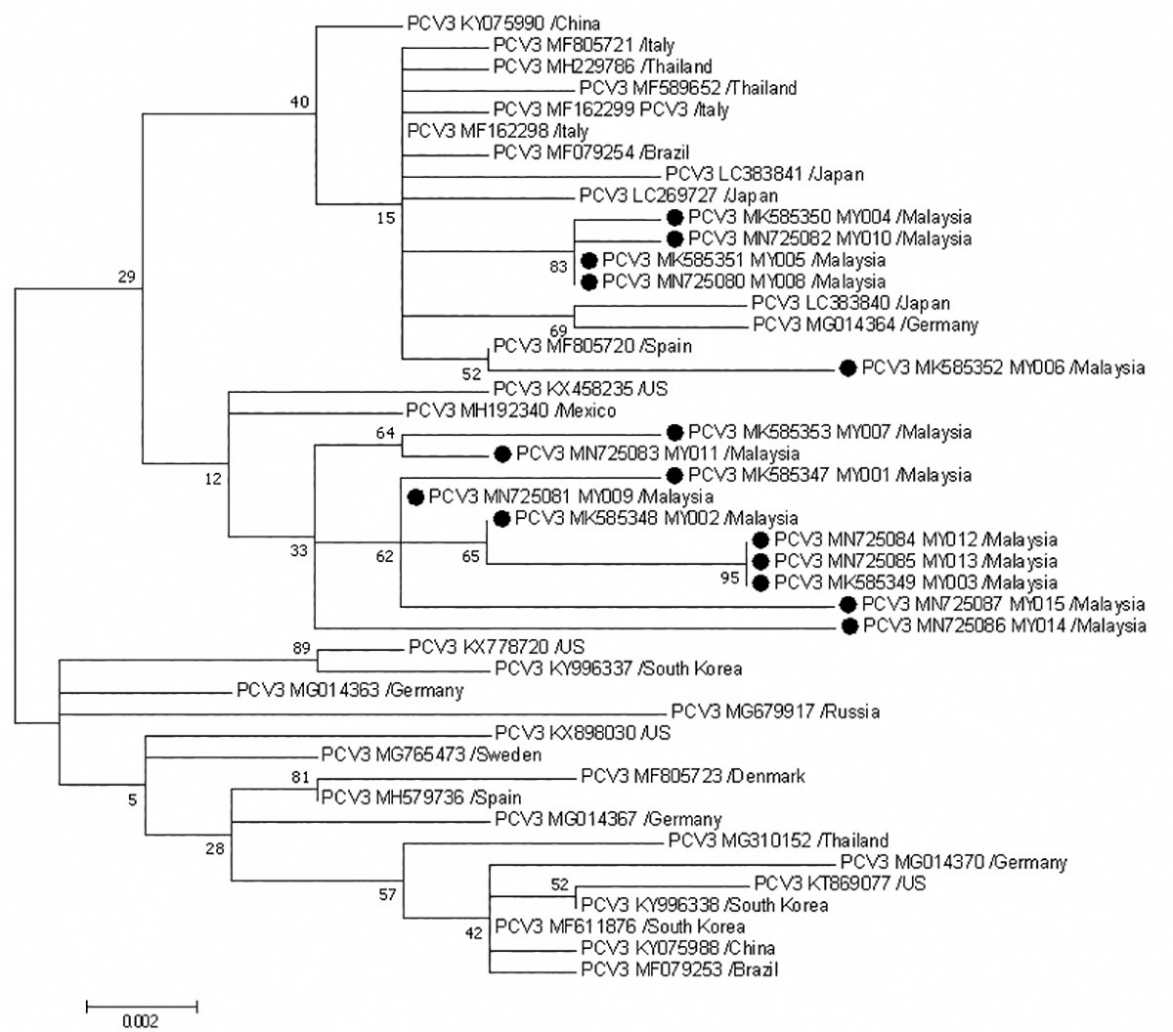
Phylogenetic analysis of *cap* gene sequences of PCV3. Fifteen Malaysian PCV3 strains (●) were compared with 30 other PCV3 strains. GenBank accession numbers and origin country are as indicated. Malaysian PCV3 strains were additionally labelled with strain ID. The tree was constructed using ML method, Tamura-Nei model evaluated with 1000 bootstrap replicates. The scale bar indicates branch length measured in number of substitutions per site.

Based on the phylogenetic analysis of PCV3 *cap* gene sequences (Fig 4), Malaysian PCV3 strains appear to be grouped into two main clusters within one clade. In the first cluster, Malaysian PCV3 strains were grouped with PCV3 strains from Italy (GenBank accession no.: MF805721, MF162298, MF162299), Thailand (MF589652, MH229786), Brazil (MF079254), Japan (LC269727, LC383840, LC383841), Germany (MG014364) and Spain (MF805720). Particularly, Malaysian strain MY006 (MK585352) showed high bootstrap value of >50% with a Spanish strain. The second cluster consisted entirely of Malaysian PCV3 strains, with only one U.S. strain (KX458235) and one Mexico strain (MH192340).

To determine positive and/or negative selections in the ORF1 and ORF2 genes of PCV3 sequences, neutrality test values, dN–dS values and H(x) entropy values of the two genes were evaluated. Results from the statistical tests of neutrality ran on PCV3 ORF1 and ORF2 gene sequences were summarized in Table 5. All three tests showed negative neutrality values of statistical significance.

**Table 5:** Results tabulation of PCV3 ORF1 and ORF2 tests of neutrality.

Gene Sequence	Tests of Neutrality					
	Tajima’s D		Fu and Li’s D		Fu and Li’s F	
	Test statistic	Statistical significance, p-value	Test statistic	Statistical significance, p-value	Test statistic	Statistical significance, p-value
ORF1	-2.41372	< 0.01	-3.86411	< 0.02	-3.98503	< 0.02
ORF2	-2.04422	< 0.05	-3.75641	< 0.02	-3.73811	< 0.02

**Fig 5:**
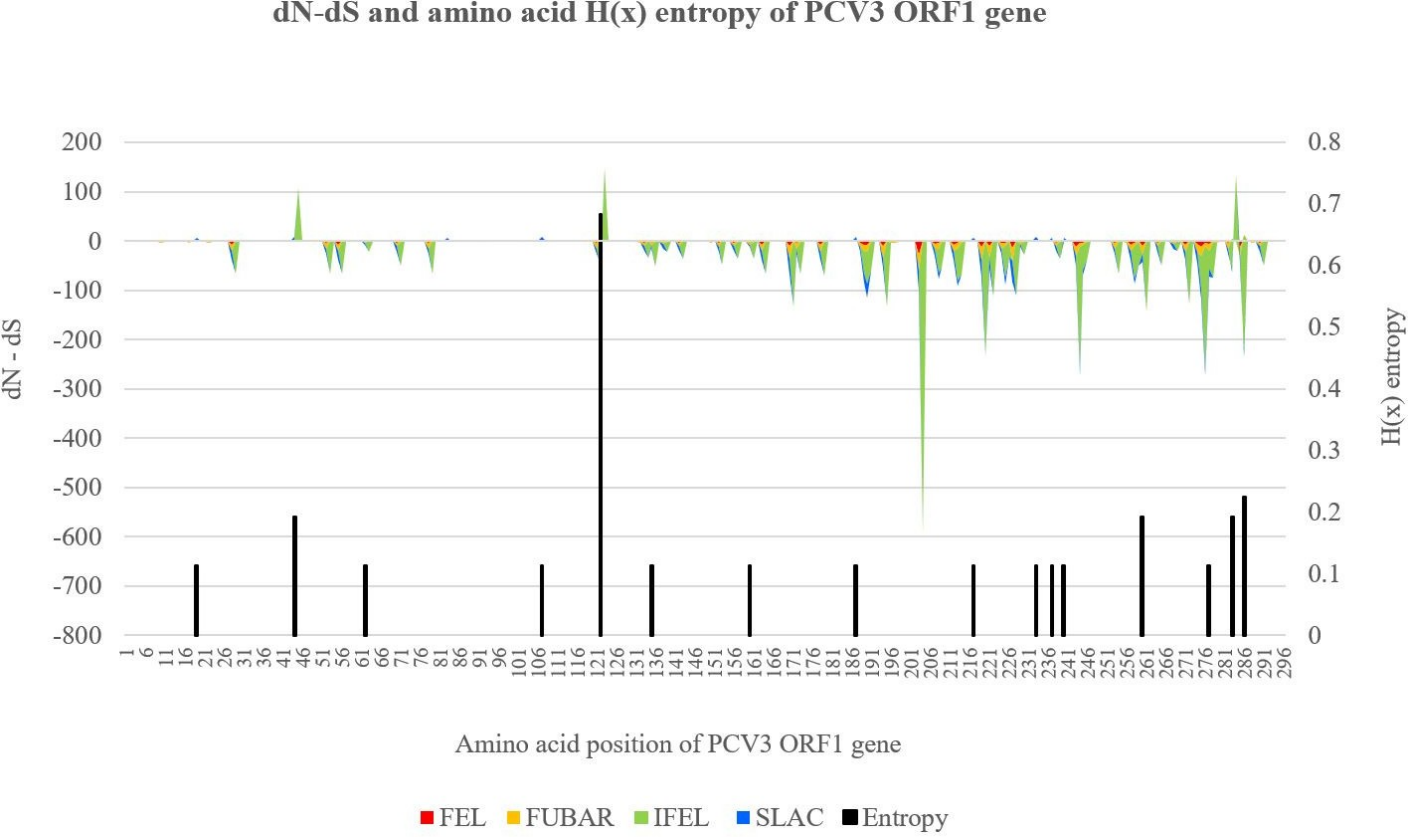
dN−dS and amino acid H(x) entropy values plot for PCV3 ORF1 gene sequences. Left y-axis corresponds to the dN–dS values of the primary stacked line graph, where values obtained by four different selection pressure methods were identified with four different primary colours. Right y-axis corresponds to H(x) entropy values of the secondary bar chart. X-axis marks the aa sites of the ORF1 gene sequence. The graphs in Figs 5 and 6 were scaled identically.

**Fig 6:**
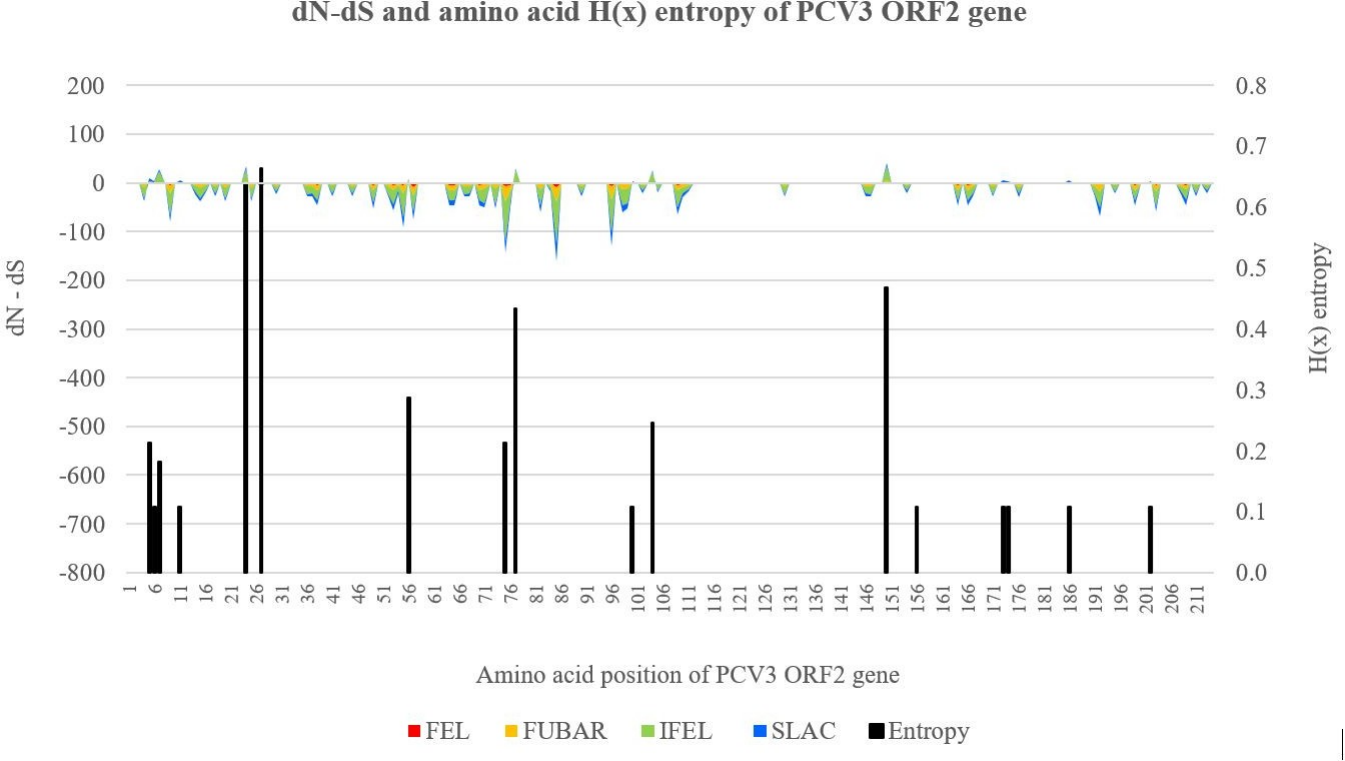
dN−dS and amino acid H(x) entropy values plot for PCV3 ORF2 gene sequences. Left y-axis corresponds to the dN–dS values of the primary stacked line graph, where values obtained by four different selection pressure methods were identified with four different primary colours. Right y-axis corresponds to H(x) entropy values of the secondary bar chart. X-axis marks the aa sites of the ORF2 gene sequence. The graphs in Figs 5 and 6 are scaled identically.

H(x) entropy values for each aa position of PCV3 ORF1 and ORF2 are plotted in the secondary bar charts of Figs 5 and 6 respectively. Among the ORF1 gene sequence, 5.41% (16 / 296) of aa sites showed H(x) entropy values of > 0.0. The 16 H(x) values ranged from 0.113 to 0.683, with a mean of 0.170 ± 0.036. ORF2 gene sequences have slightly more aa sites with H(x) entropy > 0.0, at 7.94% (17 / 214). The 17 H(x) values ranged from 0.107 to 0.663, with a mean of 0.247 ± 0.046.

Analysis of the selective pressures revealed minor differences between ORF1 and ORF2 genes at both global and site levels. Global dN–dS value of ORF1 and ORF2 were 0.015 and 0.091 respectively, as determined by SLAC method. At site levels, dN–dS values for every codon were determined to identify aa positions under positive or negative selection pressure, as tabulated in Table 6. Large majority of the calculated dN–dS values were < 0, indicating negative selection. At the statistical significance thresholds applied in this study, no positive selection was reported. Within the ORF1 and ORF2 gene, 24 / 296 (8.11%) and 26 / 214 (12.15%) aa sites respectively were identified as negatively selected sites. Among the four selection pressure methods used, descending detection rates were observed from FUBAR, FEL, SLAC to IFEL method. Only 3 sites, codon 203 of the ORF1 gene, codon 75 and 85 of the ORF2 gene, were identified as negative selection sites across all four methods.

**Table 6:** Codon selection pressures on amino acid sites of PCV3 ORF1 and ORF2.

ORF1 (*rep* gene)												ORF2 (*cap* gene)											
Site	FEL (dN–dS)	p-value	IFEL (dN–dS)	p-value	SLAC (dN–dS)	p-value	FUBAR (dN–dS)	Posterior probability	MEME (dN/dS)	p-value	Statistical method consensus (n)	Site	FEL (dN–dS)	p-value	IFEL (dN–dS)	p-value	SLAC (dN–dS)	p-value	FUBAR (dN–dS)	Posterior probability	MEME (dN/dS)	p-value	Statistical method consensus (n)
28	-8.337	0.051	0.000	1.000	-17.871	0.207	**-12.027**	**0.911**	0.018	0.670	1	9	**-4.885**	**0.013**	-46.208	0.122	**-13.110**	**0.091**	**-15.807**	**0.990**	0.117	0.670	3
55	**-8.456**	**0.050**	0.000	1.000	-17.965	0.196	**-12.134**	**0.909**	0.015	0.670	2	38	**-5.990**	**0.043**	-21.962	0.285	-9.710	0.224	**-8.683**	**0.909**	0.067	0.670	2
78	-4.772	0.078	0.000	1.000	-10.546	0.333	**-10.729**	**0.907**	0.101	0.670	1	49	**-4.965**	**0.021**	-23.684	0.234	**-12.778**	**0.086**	**-10.704**	**0.981**	0.101	0.670	3
121	-4.890	0.089	0.000	1.000	-10.546	0.333	**-10.926**	**0.904**	0.092	0.670	1	53	**-4.362**	**0.029**	-28.375	0.224	-11.220	0.111	**-11.830**	**0.983**	0.128	0.670	2
163	-8.344	0.063	0.000	1.000	-17.934	0.227	**-12.018**	**0.907**	0.014	0.670	1	55	**-4.647**	**0.011**	-49.553	0.120	**-21.439**	**0.034**	**-16.465**	**0.998**	0.116	0.670	3
170	**-10.155**	**0.020**	0.000	1.000	**-31.639**	**0.048**	**-26.546**	**0.997**	0.000	0.670	3	57	**-8.992**	**0.004**	-33.177	0.171	**-17.495**	**0.046**	**-15.624**	**0.990**	0.028	0.670	3
178	**-8.838**	**0.048**	0.000	1.000	-17.538	0.211	**-12.505**	**0.913**	0.012	0.670	2	64	**-5.930**	**0.035**	-21.793	0.275	-9.691	0.193	**-8.628**	**0.912**	0.074	0.670	2
189	-8.227	0.064	-47.052	0.339	-17.872	0.228	**-11.877**	**0.906**	0.016	0.670	1	65	**-5.917**	**0.045**	-21.793	0.251	-9.691	0.193	**-8.624**	**0.910**	0.064	0.670	2
190	**-7.466**	**0.018**	-64.882	0.295	**-28.201**	**0.062**	**-14.23**	**0.983**	0.032	0.670	3	70	**-5.929**	**0.032**	-21.802	0.281	-9.691	0.193	**-8.633**	**0.912**	0.079	0.670	2
194	**-9.904**	**0.027**	0.000	1.000	-21.092	0.111	**-23.947**	**0.992**	0.006	0.670	2	71	-2.998	0.051	-30.396	0.187	-8.722	0.218	**-8.511**	**0.915**	0.237	0.670	1
203	**-28.651**	**0.004**	**-591.414**	**0.027**	**-39.881**	**0.040**	**-32.089**	**0.996**	0.000	0.670	4	73	**-3.099**	**0.049**	-31.292	0.183	-8.802	0.216	**-8.767**	**0.917**	0.229	0.670	2
208	-4.637	0.077	-50.053	0.304	-12.299	0.329	**-10.508**	**0.966**	0.099	0.670	1	75	**-7.484**	**0.020**	**-83.637**	**0.022**	**-22.428**	**0.046**	**-29.419**	**0.989**	0.168	0.670	4
212	-8.221	0.064	0.000	1.000	-17.719	0.229	**-11.875**	**0.906**	0.0158	0.670	1	76	**-4.740**	**0.024**	-22.087	0.249	**-12.070**	**0.096**	**-10.055**	**0.978**	0.110	0.670	3
219	**-12.379**	**0.017**	0.000	1.000	**-34.335**	**0.054**	**-25.356**	**0.993**	0.000	0.670	3	82	**-3.048**	**0.029**	-32.518	0.176	**-14.240**	**0.077**	**-10.571**	**0.981**	0.230	0.670	3
221	**-9.682**	**0.012**	0.000	1.000	-24.609	0.108	**-22.337**	**0.998**	0.000	0.670	2	85	**-8.198**	**0.000**	**-87.539**	**0.029**	**-35.932**	**0.002**	**-29.124**	**1.000**	0.037	0.670	4
227	**-14.120**	**0.005**	0.000	1.000	**-42.946**	**0.020**	**-25.231**	**0.998**	0.000	0.670	3	96	**-6.907**	**0.004**	-68.891	0.056	**-28.051**	**0.004**	**-26.277**	**1.000**	0.038	0.670	3
243	**-12.271**	**0.042**	0.000	1.000	-19.919	0.191	**-17.718**	**0.937**	0.000	0.670	2	98	**-3.022**	**0.038**	-32.233	0.207	-14.253	0.106	**-10.478**	**0.979**	0.225	0.670	2
253	-4.996	0.075	0.000	1.000	-10.546	0.333	**-11.489**	**0.913**	0.090	0.670	1	99	-3.065	0.078	-30.977	0.251	-8.782	0.264	**-8.654**	**0.908**	0.219	0.670	1
257	-8.344	0.063	0.000	1.000	-17.934	0.227	**-12.018**	**0.907**	0.014	0.670	1	109	**-4.260**	**0.050**	-35.045	0.180	-14.789	0.126	**-10.349**	**0.924**	0.141	0.670	2
271	-7.898	0.059	0.000	1.000	-15.615	0.226	**-15.186**	**0.927**	0.020	0.670	1	110	**-3.495**	**0.042**	-12.861	0.331	-8.051	0.232	-5.068	0.881	0.201	0.670	1
274	**-6.698**	**0.031**	0.000	1.000	-21.092	0.111	**-16.482**	**0.986**	0.040	0.670	2	164	**-5.881**	**0.045**	-21.609	0.252	-9.672	0.193	**-8.550**	**0.910**	0.065	0.670	2
275	**-12.365**	**0.042**	-69.403	0.215	-19.961	0.191	**-17.762**	**0.937**	0.000	0.670	2	166	**-5.917**	**0.045**	-21.793	0.251	-9.691	0.193	**-8.623**	**0.910**	0.064	0.670	2
277	-5.142	0.247	-35.504	0.410	-15.819	0.259	**-15.862**	**0.925**	0.266	0.670	1	192	**-3.360**	**0.039**	-34.412	0.167	**-16.831**	**0.037**	**-13.208**	**0.996**	0.185	0.670	3
285	**-12.098**	**0.043**	0.000	1.000	-19.899	0.192	**-17.618**	**0.937**	0.000	0.670	2	199	**-5.969**	**0.024**	-21.961	0.236	-9.710	0.193	**-8.703**	**0.917**	0.087	0.670	2
												203	**-4.874**	**0.024**	-30.061	0.214	-11.220	0.111	**-12.632**	**0.985**	0.102	0.670	2
												209	**-4.175**	**0.020**	-20.208	0.268	-11.220	0.111	**-9.486**	**0.977**	0.141	0.670	2
Method		FEL	IFEL	SLAC	FUBAR	MEME	Statistical method consensus (n)		n = 1	n = 2	n = 3	n = 4	Method		FEL	IFEL	SLAC	FUBAR	MEME	Statistical method consensus (n)	n = 1	n = 2	n = 3	n = 4
Positively Selected Sites		0	0	0	0	0	Positively Selected Sites		0	0	0	0	Positively Selected Sites		0	0	0	0	0	Positively Selected Sites	0	0	0	0
Negatively Selected Sites		13 / 296 (4.39%)	1 / 296 (0.34%)	5 / 296 (1.69%)	24 / 296 (8.11%)	N/A	Negatively Selected Sites		11 / 296 (3.72%)	8 / 296 (2.70%)	4 / 296 (1.35%)	1/296 (0.34%)	Negatively Selected Sites		24 / 214 (11.21%)	2 / 214 (0.93%)	10 / 214 (4.67%)	25 / 214 (11.68%)	N /A	Negatively Selected Sites	3 / 214 (1.40%)	13 / 214 (6.07%)	8 / 214 (3.74%)	2 / 214 (0.93%)
							Total		24 / 296 (8.11%)											Total	26 / 214 (12.15%)			
							Global dN–dS (SLAC)		0.115											Global dN–dS (SLAC)	0.091	

Comparison between dN and dS is expressed as dN–dS (dN / dS for MEME). Values of dN–dS < 0, = 0 and > 0 (dN / dS < 1, = 1 and > 1 for MEME method) indicates negative selection, neutral evolution and positive selection respectively. Significance level was set to p-value ≤ 0.1 for SLAC and MEME method, p-value ≤ 0.05 for FEL and IFEL methods, posterior probability > 0.9 for FUBAR method. dN–dS values of statistical significance are highlighted in boldface. Statistical method consensus (n) indicates number of different test methods generating significant value for one same aa site.

## Discussion

PCV3 has been detected in various tissue samples with varying positive rates [15, 34–38]. Our findings are in agreement with reports from Fan *et al*. (2017), Fu *et al*. (2018) and Li *et al*. (2018), that PCV3 mainly infect lungs and lymphoid tissues such as lymph nodes and tonsil [34, 35, 38]. PCV2 is known to have tropism for lymphoid tissues in pigs, with lymphoid depletion and histiocytic replacement of lymphoid tissues being hallmark lesions of PCV2 infection [39, 40]. Interstitial pneumonia and bronchiolitis with mononuclear infiltration are also part of PCV2 disease manifestation, which correlate with clinical sign of respiratory distress [41, 42]. Since high molecular detection rates of 81.82% and 71.43% respectively were found in inguinal lymph nodes and lungs, with demonstrated statistical significance in molecular detection rate of PCV3 in the inguinal lymph nodes group, further research may be focused on these tissues to study potential tissue tropism and relationship with PCV3 pathogenesis.

Weaners have been shown to have the highest prevalence of PCV3 [7, 43, 44] across different production stages and statistical results of this study supports this finding. PCV3 has been reported in pigs with various pathological conditions including respiratory, reproductive, neurological and gastrointestinal disorders. Statistics calculated in this study also showed high PCV3 molecular detection rate in the clinically ill group. Nevertheless, the virus has also been detected in clinically healthy animals [45–47]. However, in this study, all lungs samples of clinically healthy pigs sourced from abattoir and retail shops were tested negative for PCV3 in spite of their origins in PCV3 positive farms. This 100% negative results may not be accurate, considering the low sample number which constitute only 12.76% (18 / 141 pigs) of the study population, and that sampling healthy animals solely from finishers stage may not be representative for the clinically healthy population.

Apart from domestic pigs, PCV3 was found to infect wild boar population at rates of 23%– 42.66% [37, 48, 49]. Notably, all 28 lung samples from wild boar population in Peninsular Malaysia were tested negative for PCV3. This finding may suggest that spillover infection from wild boar reservoir hosts is not implicated in introduction of PCV3 into Malaysian commercial swine population. PCV3 infection susceptibility has been suggested to be associated with the age of wild boar, with juvenile animals showing statistically lower detection rates, unlike reports described in domestic pigs [37, 48]. In our study, 46.42% (13 / 28 animals) were identified as adults of > 12 months old, with another 17.86% (5 / 28 animals) categorized as 6–12 months old subadults, thus eliminating the concern of age group bias. High PCV3 prevalence of 33.15% was reported by Franzo *et al*. [48], despite sampling mostly apparently healthy wild boars (60 / 62 animals), suggesting that PCV3 may be unlikely to cause overt clinical diseases in wild boars. If such is the case, serum samples might be more suitable for study of PCV3 prevalence in wild boar population, as compared to lung samples used in this study. Nevertheless, 57.14% (20 / 35 samples) and 54.29% (19 / 35 samples) detection rates in lung and spleen samples respectively have been reported [37]. Possibility of false negative prevalence of PCV3 in wild boar population in this study due to low sample number still need to be considered.

PCV3 sequences from different years and countries analysed to date showed nt similarities ranging from 97 to 100% [50]. The complete genome sequences of Malaysian PCV3 strains in this study showed similarities of 97.9%– 99.8%, concurring with the summarized global findings. In the case of PCV2, ORF1 is the most conserved region of circovirus genome spanning the entire genome sequence [51]. In contrast, ORF2 has been identified as the most variable [6] and most immunogenic viral protein [52, 53]. The assumption that the PCV3 *cap* gene is also a variable region like its PCV2 counterpart was supported as the 45 PCV3 *cap* gene sequences analyzed in this study showed higher p-distance values, when compared to the complete genomes in pairwise distance analysis. This indicates that the *cap* genes show higher variability than the complete genomes, in terms of number of base differences per site.

Tajima’s D, Fu and Li’s D, and Fu and Li’s F statistical tests of neutrality share a common foundation of utilizing the frequency distribution of mutations. The unanimous negative values of all three neutrality tests signify an excess of low frequency polymorphisms relative to expectation, an effect resulting from either purifying selection or expansion of population size [25, 26]. Considering the general negative trend of dN–dS values as shown in Figs 5 and 6, we suggest that the ORF1 and ORF2 genes of PCV3 may be heavily influenced by negative selection pressure. In this study, up to 12.15% (26 / 214) of ORF2 codons were predicted to be evolving under negative selection pressure. ORF1 has a lower percentage of 8.11% (24 / 296). A recent study predicted up to 17% (37 / 214) of PCV3 ORF2 codons were negatively selected and suggested a strong negative selection acting over ORF2 gene of PCV3, which will likely cause the gene to be subjected to strong restrictions and thus unable to tolerate high levels of variation on its sequence [54]. In the context of diversity of aa residues at a given position, entropy H(x) values range from 0.0 (single residue present) to 4.322 (all 20 residues equally represented). As the H(x) value increases, it would be more likely to observe different aa residues diversity at same codon position. Aa with H(x) ≥ 2.0 are considered variable, while highly conserved aa would be expected to have H(x) of ≤ 1.0 [55]. For PCV3, just as ORF2 has higher dN–dS values as compared to ORF1, ORF2 also have slightly more aa sites with H(x) entropy > 0.0, at 7.94% (17 / 214). This again suggests that ORF2 has higher genetic variability, in terms of number of base differences per site. However, all the H(x) values were well below ≤ 1.0, supporting the conjecture of PCV3 ORF2 being under strong purifying selection processes [53]. The highest H(x) value of ORF1 was seen at aa position 122, with a value of 0.683. The highest H(x) values of ORF2 ranged from 0.432–0.663, seen at aa position 24, 27, 77 and 150. The aa substitutions resulting from nt mutations at these five codons were suggested as criteria to classify PCV3 into different strains [7]. Apart from being a component of classification criteria, ORF2 aa position 24 was the convergence of several studies, proposed as a potential epitope region and determinant of antigen effect [38, 56–58].

Since the *cap* genes are much more divergent, differences over nt sequences may be resolved by phylogenetic comparison of the *cap* gene coding sequence, ORF2. Hence, the genomic variability of Malaysian PCV3 strains were compared by having their 645 bp long *cap* gene sequences phylogenetically analysed. Though the Malaysian PCV3 strains are rather closely related to each other, as reflected by p-distance values of < 0.021, they were grouped into two distinct clades.

This study suggests a homogeneous PCV3 frequency among farms with different size of standing sow population and proximity with neighbouring farms, since no statistically significant differences were found among those tested groups. There was no apparent geographical distribution, strains from central and southern Malaysia are present in both clades. However, it was observed that strains from the same farm are closely related, as shown by strains MY008 (GenBank Accession no.: MN725080) and MY010 (MN725082); MY001 (MK585347), MY002 (MK585348), MY009 (MN725081), MY012 (MN725084) and MY013 (MN725085). Interestingly, from the same farm, strains MY012 and MY013 which were obtained on a later time point in 2018/2019 showed further phylogenetic distance from strains MY001, MY002 and MY009 obtained in 2016/2017. In another farm, a phylogenetic distance gap was seen between strain MY008 (MN725080) and strain MY010 (MN725082) collected five months apart. This may suggest that PCV3 may have the tendency to mutate rapidly, as what was seen with PCV2. High mutation rates of PCV2 were widely reported [59–61]. Over the years, new PCV2 antigenic variants have evolved with the variability of PCV2 attributed to high evolutionary rates of 1.2x10^−3^ substitution/site/year, higher than expected for a DNA virus and instead resembling RNA viruses [62]. In addition, low frequency mutations that allow rapid adaptation of the virus in changing environments had been identified in PCV2 genomes [60].

In the first clade, notably, Malaysian strain MY006 (MK585352) was singled out in its own cluster with a Spanish strain (MF805720), at a high bootstrap value of 52%. The singling out of MY006 strain may be attributed to the isolated location and strict biosecurity practice of the origin farm that possibly reduced the likelihood of introducing circulating PCV3 strains from other local farms. Given that PCV3 can be found in semen of healthy animals [15] and that potential persistent infection nature of PCV3 has been suggested [37], the phylogenetic relationship between Malaysian and Spanish strains might be related to semen and breeder importation. Malaysian PCV3 strains also showed phylogenetic relationship with PCV3 strains from Italy, Thailand, Brazil, Japan and Germany, though with lower bootstrap values. However, of these countries, only Thailand and Germany have trade activities involving porcine products with Malaysia in the past 10 years [63]. In the second clade, Malaysian PCV3 strains were closely related to a U.S. strain (KX458235) and a Mexico strain (MH192340). Since 2001, Malaysia imports live breeders from U.S., from 30 to 200 heads annually though not consistently [63]. U.S. is also the main provider of live swine imports for Mexico, with a 72% share of the Mexican import market [64]. Hence, it may be speculated that the phylogenetic relatedness among Malaysian, Spanish, U.S. and Mexico PCV3 strains might be a result of live breeder and semen stock movement.

The first and second clades discussed above are evidently grouped together in a larger cluster. This observation is in accordance with the PCV3 strain classification system proposed by Fux *et al*. [7]. Fux *et al*. observed a specific aa motif which divides the PCV3 strains into two main groups. On aa position 122 of ORF1; aa position 24, 27, 77, 150 of ORF2 and aa position 1, 4, 227 of ORF3, two distinct patterns were observed: A–V K S I–F D G and S–A R S I–S G V, which defined group A1 and B1 respectively. Slight modification of motif into S–V K S I–F D V and S–A R T L–S G V gave rise to subgroups A2 and B2 respectively. Based on extrapolation from Fux *et al*.’s categorization, Malaysian PCV3 strains were classified as PCV3 group A1 and A2 (Table 7).

**Table 7:** Amino acid alignments of selected aa in ORFs 1, 2 and 3 showing group specific motifs.

ORF		ORF1	ORF2				ORF3		
Strain		122	24	27	77	150	1	4	227
KY075990/China	**A1**	A	V	K	S	I	F	D	G
MF805721/Italy	**A1**	A	V	K	S	I	F	D	G
MH229786/Thailand	**A1**	A	V	K	S	I	F	D	G
MF589652/Thailand	**A1**	A	V	K	S	I	F	D	G
MF162299/Italy	**A1**	A	V	K	S	I	F	D	G
MF162298/Italy	**A1**	A	V	K	S	I	F	D	G
MF079254/Brazil	**A1**	A	V	K	S	I	F	D	G
LC383841/Japan	**A1**	A	V	K	S	I	F	D	G
LC269727/Japan	**A1**	A	V	K	S	I	F	D	G
PCV3/SL52/UPM/MY004/MK585350	**A1**	A	V	K	S	I	F	D	G
PCV3/SW35L/UPM/MY010/MN725082	**A1**	A	V	K	S	I	F	D	G
PCV3/SL56/UPM/MY005/MK585351	**A1**	A	V	K	S	I	F	D	G
PCV3/SW13L/UPM/MY008/MN725080	**A1**	A	V	K	S	I	F	D	G
LC383840/Japan	**A1**	A	V	K	S	I	F	D	G
MG014364/Germany	**A1**	A	V	K	S	I	F	D	G
MF805720/Spain	**A1**	A	V	K	S	I	F	D	G
PCV3/JL72/UPM/MY006/MK585352	**A2**	S	V	K	S	I	F	D	V
KX458235/US	**A2**	S	V	K	S	I	F	D	V
MH192340/Mexico	**A2**	S	V	K	S	I	F	D	V
PCV3/KG11/UPM/MY007/MK585353	**A2**	S	V	K	S	I	F	D	V
PCV3/PG43i/UPM/MY011/MN725083	**A2**	S	V	K	S	I	F	D	V
PCV3/SL28/UPM/MY001/MK585347	**A2**	S	V	K	S	I	F	D	V
PCV3/SS31L/UPM/MY009/MN725081			V	K	S	I			
PCV3/SL37/UPM/MY002/MK585348	**A2**	S	V	K	S	I	F	D	V
PCV3/SW51L/UPM/MY012/MN725084	**A2**	S	V	K	S	I	F	D	V
PCV3/SW53L/UPM/MY013/MN725085	**A2**	S	V	K	S	I	F	D	V
PCV3/KL44/UPM/MY003/MK585349			V	K	S	I			
PCV3/KG194L/UPM/MY/015/MN725087			V	K	S	I			
PCV3/MG196L/UPM/MY014/MN725086	**A2**	S	V	R	S	I	S	D	V
KX778720/US	**B1**	A	A	R	S	I	S	G	G
KY996337/Korea	**B1**	A	V	R	S	I	S	D	G
MG014363/Germany	**B1**	S	A	R	S	I	S	G	V
MG679917/Russia	**B1**	S	A	R	S	L	S	G	V
KX898030/US	**B1**	S	A	R	S	I	S	G	V
MG765473/Sweden	**B1**	S	A	R	S	I	S	G	V
MF805723/Denmark	**B1**	S	A	R	S	I	S	G	V
MH579736/Spain	**B1**	S	A	R	S	I	S	G	V
MG014367/Germany	**B1**	S	A	R	S	I	S	G	F
MG310152/Thailand	**B2**	S	A	R	T	L	S	G	V
MG014370/Germany	**B2**	S	A	R	T	L	S	G	V
KT869077/US	**B2**	S	A	R	T	L	S	G	V
KY996338/Korea	**B2**	S	A	R	T	L	S	G	V
MF611876/Korea	**B2**	S	A	R	T	L	S	G	V
KY075988/China	**B2**	S	A	R	T	L	S	G	V
MF079253/Brazil	**B2**	S	A	R	T	L	S	G	V

Similarly, there was no geographical distribution pattern observed, for both Malaysian and global strains of PCV3 A1, A2, B1 and B2. The arrangement of the motif was reflected in the phylogenetic clade arrangement, with strain A and B each clustered into separate clade (Fig 4). Further clustering into strain 1 and 2 were more evident in the A group, where clade A1 and A2 were clearly branched apart. However, more PCV3 sequences are needed to validate this genotype classification, and further work is required to determine if these genetic differences correlate to specific biological properties of PCV3 [7]. Nevertheless, with the rapid emergence of PCV2 genotype 2d and 2e unfurling the speed of worldwide distribution of a new type of PCV [65], these phylogenetic and epidemiological data may give us a head start with PCV3.

In terms of relationship with other species of *Circovirdae*, China strains of PCV3 was reported to share a clade with bat associated circovirus 8 (BatACV8) at a high 82% bootstrap value [38]; while the U.S. strains of PCV3 traced back to a common ancestor bat associated circovirus 2 (BatACV2) [4]. Malaysian strains of PCV3 showed phylogenetic relatedness to bat-associated circovirus 7 (BatACV7) and StCV with a 30% bootstrap value. Another instance of involvement of bats in porcine diseases would be fruit bats of *Pteropid* species acting as natural reservoir hosts of Nipah virus, where a Nipah outbreak in the 1990s almost costed the entire Malaysian swine industry [66, 67]. As for starlings (*Sturnus vulgaris*), they are native to Europe, Asia and North Africa and have successfully established populations on nearly every continent [68]. Starling fecal material has been shown to be one of the transmission sources of transmissible gastroenteritis (TGE) coronavirus, with history of TGE outbreaks in swine farms attributed to starlings as outbreak vector [69]. Although *Circovirus* genus members are known to infect a wide host range [33], cases of unspecific cross-species transmission had been reported. PCV1 and PCV2 has been detected human stool samples, and avian circovirus-like DNA was found in wild chimpanzee feces samples [14]. PCV3 has also been detected in chamois, roe deer [70], cattle [71] and canine [72]. Considering the possibility of cross-species transmission, documented pathogenicity of closely related *Circovirus* species, high mutation and recombination rate of certain ssDNA viruses [14], and history of bats and starlings transmitting porcine pathogens, further investigation into the relationship between PCV3 with BatACV and StCV may be warranted. On the other hand, the most recently discovered PCV4, showed closest genomic and phylogenetic relationship with mink circovirus (MiCV) [3]. The pathogenicity of PCV4 remains unclear and to date, no direct relationship between PCV3 and PCV4 has been reported.

## Conclusion

PCV3 is present in Peninsular Malaysia at a molecular prevalence of 17.02%, with inguinal lymph nodes and lungs showing the highest molecular detection rates of 81.82% and 71.43% respectively. Among the nine organ types tested, only the molecular detection rate in inguinal lymph nodes was statistically significant. Although PCV3 positive samples spanned across all age group from foetuses to finishers and sows, only the weaners group was shown to be statistically significant. Despite wide reports of PCV3 in healthy animals and wild boars, no positive samples were detected in the clinically healthy finishers and wild boar population in this study. PCV3 strains included in this study were found to be heavily influenced by negative selection pressure. In both ORF1 and ORF2, aa positions with the highest H(x) values correspond with the distinct mutation patterns included in the current PCV3 strain classification system. Malaysian PCV3 strain A1 and A2 were phylogenetically related to Spanish, U.S. and Mexico strains.

## Supporting information

S1 Table Characteristics of commercial swine farms sampled in this study. (DOCX)

S2 Table Result tabulation of PCV3 PCR detection. Origin (Farm), collection year, clinical health status, age group and organ types collected for each sampled animal is detailed as follows. Lung, inguinal lymph node, spleen, tonsil, kidney, heart, mesenteric lymph node, liver and brain were sampled if available. Organ samples with positive or negative PCR results for PCV3 are indicated with ‘+’ or ‘N’ respectively. Organ samples that were not collected or not tested are indicated as ‘N/A’ (Not available). The 14 animals with sufficient organ sample types that were included in the molecular detection rate comparison are indicated with yellow highlight and boldface.
(DOCX)

S3 Table Detailed statistical calculation for Chi-square and Fisher’s exact tests evaluating association between PCV3 molecular detection status and age group, health status, farm standing sow population, distance from neighbouring farms, and across different organs. Statistically significant values are highlighted with grey boxes and boldface.
(DOC)

S4 Table Pairwise distance analysis of complete genomes of PCV3, shown as p-distance values. Twelve Malaysian PCV3 strains and 30 PCV3 GenBank reference strains were compared. The analysis was run using Pairwise Distance method, p-distance method evaluated with 1000 bootstrap replicates. P-distance values of ≥ 0.020 are indicated in grey boxes.
(DOCX)

S5 Table Pairwise distance analysis of complete genomes of PCV3, shown as percentage nucleotide identities. Twelve Malaysian PCV3 strains and 30 PCV3 GenBank reference strains were analysed for p-distance values as described in S4 Table. p-distance values are represented as percentage nt identities here in S5 Table. Percentage nt identities of ≤ 98.05% are indicated in grey boxes.
(DOCX)

S6 Table Pairwise distance analysis of *cap* gene sequences of PCV3, shown as p-distance values. Fifteen Malaysian PCV3 strains and 30 PCV3 GenBank reference strains were compared. The analysis was run using Pairwise Distance method, p-distance method evaluated with 1000 bootstrap replicates. P-distance values of ≥ 0.020 are indicated in grey boxes.
(DOCX)

S7 Table Pairwise distance analysis of *cap* gene sequences of PCV3, shown as percentage nucleotide identities. Fifteen Malaysian PCV3 strains and 30 PCV3 GenBank reference strains were analysed for p-distance values as described in S6 Table. P-distance values are represented as percentage nt identities here in S7 Table. Percentage nt identities of ≤ 97.36% are indicated in grey boxes.
(DOCX)
