# Supplementary material for: First molecular detection and complete sequence analysis of porcine circovirus type 3 (PCV3) in Peninsular Malaysia
Source: PLoS One. 2020 Jul 24;15(7):e0235832. doi: 10.1371/journal.pone.0235832 (PMC7380639; doi:10.1371/journal.pone.0235832)
Supplement: S1 Table — (DOCX) [file pone.0235832.s001.docx]

**Supplementary Table 1. Characteristics of commercial swine farms sampled in this study.**

| **Farm ID** | **State / Region in Malaysia** | **Farm Size  (Sow Number)** | **Distance from Neighbouring Swine Farm** |
| --- | --- | --- | --- |
| S1 | Selangor / Central | 1000 | <1 km |
| S2 | Selangor / Central | 1200 | <1 km |
| S3 | Selangor / Central | 300 | <1 km |
| S4 | Selangor / Central | 200 | <1 km |
| S5 | Selangor / Central | 200 | <1 km |
| S6 | Selangor / Central | 100 | <1 km |
| S7 | Selangor / Central | 400 | <1 km |
| S8 | Selangor / Central | 300 | <1 km |
| S9 | Selangor / Central | 800 | <1 km |
| K1 | Perak / Northern | 400 | > 10 km |
| K2 | Perak / Northern | 400 | < 1 km |
| K3 | Perak / Northern | 4500 | 1 – 10 km |
| K4 | Perak / Northern | 760 | 1 – 10 km |
| K5 | Perak / Northern | 300 | 1 – 10 km |
| M1 | Melaka / Southern | 500 | < 1km |
| M2 | Melaka / Southern | 600 | 1 – 10 km |
| M3 | Melaka / Southern | 180 | <1 km |
| J1 | Johor / Southern | 500 | >10 km |
| J2 | Johor / Southern | 2000 | >10 km |
| J3 | Johor / Southern | 1700 | >10 km |
| P1 | Penang / Northern | 300 | <1 km |
| P2 | Penang / Northern | 200 | <1 km |
| P3 | Penang / Northern | 200 | <1 km |
| P4 | Penang / Northern | 200 | <1 km |
