# Supplementary material for: First molecular detection and complete sequence analysis of porcine circovirus type 3 (PCV3) in Peninsular Malaysia
Source: PLoS One. 2020 Jul 24;15(7):e0235832. doi: 10.1371/journal.pone.0235832 (PMC7380639; doi:10.1371/journal.pone.0235832)
Supplement: S2 Table — Origin (Farm), collection year, clinical health status, age group and organ types collected for each sampled animal is detailed as follows. Lung, inguinal lymph node, spleen, tonsil, kidney, heart, mesenteric lymph node, liver and brain were sampled if available. Organ samples with positive or negative PCR results for PCV3 are indicated with ‘+’ or ‘N’ respectively. Organ samples that were not collected or not tested are indicated as ‘N/A’ (Not available). The 14 animals with sufficient organ sample types that were included in the molecular detection rate comparison are indicated with yellow highlight and boldface. (DOCX) [file pone.0235832.s002.docx]

**Supplementary Table 2. Result Tabulation of PCV3 PCR Detection**

Origin (Farm), collection year, clinical health status, age group and organ types collected for each sampled animal is detailed as follows. Lung, inguinal lymph node, spleen, tonsil, kidney, heart, mesenteric lymph node, liver and brain were sampled if available. Organ samples with positive or negative PCR results for PCV3 are indicated with ‘+’ or ‘N’ respectively. Organ samples that were not collected or not tested are indicated as ‘N/A’ (Not available). The 14 animals with sufficient organ sample types that were included in the molecular detection rate comparison are indicated with yellow highlight and boldface.

| Pig ID | Farm  ID | Collection Year | Clinical Health Status | Age  Group | Organ Samples Collected and Tested | | | | | | | | |
| --- | --- | --- | --- | --- | --- | --- | --- | --- | --- | --- | --- | --- | --- |
|  |  |  |  |  | Lung | Inguinal lymph node | Spleen | Tonsil | Kidney | Heart | Mesenteric lymph node | Liver | Brain |
| 1 | S1 | 2016/17 | Ill | Weaner | + | N/A | N/A | N/A | N/A | N/A | N/A | N/A | N/A |
| 2 | S1 | 2016/17 | Ill | Weaner | + | N/A | N/A | N/A | N/A | N/A | N/A | N/A | N/A |
| 3 | S1 | 2018/19 | Ill | Sow | + | N/A | N/A | N/A | N/A | N/A | N/A | N/A | N/A |
| 4 | S1 | 2018/19 | Ill | Grower | N | N | N/A | N/A | N/A | N/A | N/A | N/A | N/A |
| **5** | **S1** | **2018/19** | **Ill** | **Weaner** | **+** | **+** | **+** | **+** | **+** | **N/A** | **+** | **N/A** | **N/A** |
| 6 | S1 | 2018/19 | Ill | Weaner | N | N | N/A | N/A | N/A | N/A | N/A | N/A | N/A |
| **7** | **S1** | **2018/19** | **Ill** | **Weaner** | **+** | **+** | **+** | **+** | **N** | **N/A** | **+** | **N/A** | **N/A** |
| 8 | S1 | 2018/19 | Ill | Weaner | N | N | N/A | N/A | N/A | N/A | N/A | N/A | N/A |
| 9 | S1 | 2016/17 | Ill | Weaner | N | N/A | N/A | N/A | N/A | N/A | N/A | N/A | N/A |
| 10 | S1 | 2016/17 | Ill | Weaner | N | N/A | N/A | N/A | N/A | N/A | N/A | N/A | N/A |
| 11 | S1 | 2016/17 | Ill | Weaner | N | N/A | N/A | N/A | N/A | N/A | N/A | N/A | N/A |
| 12 | S1 | 2016/17 | Ill | Weaner | N | N/A | N/A | N/A | N/A | N/A | N/A | N/A | N/A |
| 13 | S1 | 2016/17 | Ill | Weaner | N | N/A | N/A | N/A | N/A | N/A | N/A | N/A | N/A |
| 14 | S1 | 2016/17 | Ill | Weaner | N | N/A | N/A | N/A | N/A | N/A | N/A | N/A | N/A |
| 15 | S1 | 2016/17 | Ill | Weaner | N | N/A | N/A | N/A | N/A | N/A | N/A | N/A | N/A |
| 16 | S1 | 2016/17 | Ill | Weaner | N | N/A | N/A | N/A | N/A | N/A | N/A | N/A | N/A |
| 17 | S1 | 2016/17 | Ill | Weaner | N | N/A | N/A | N/A | N/A | N/A | N/A | N/A | N/A |
| 18 | S1 | 2016/17 | Ill | Weaner | N | N/A | N/A | N/A | N/A | N/A | N/A | N/A | N/A |
| 19 | S1 | 2016/17 | Ill | Weaner | N | N/A | N/A | N/A | N/A | N/A | N/A | N/A | N/A |
| **20** | **S2** | **2016/17** | **Ill** | **Weaner** | **+** | **N/A** | **+** | **N/A** | **N/A** | **N/A** | **N/A** | **N/A** | **N/A** |
| 21 | S2 | 2018/19 | Ill | Weaner | N | N | N | N/A | N/A | N/A | N/A | N/A | N/A |
| 22 | S2 | 2018/19 | Ill | Weaner | N | N | N | N/A | N/A | N/A | N/A | N/A | N/A |
| 23 | S2 | 2018/19 | Ill | Weaner | N | N | N | N/A | N/A | N/A | N/A | N/A | N/A |
| 24 | S2 | 2018/19 | Ill | Weaner | N | N | N | N/A | N/A | N/A | N/A | N/A | N/A |
| 25 | S2 | 2018/19 | Ill | Weaner | N | N | N | N | N | N/A | N/A | N/A | N/A |
| 26 | S2 | 2018/19 | Ill | Weaner | N | N | N | N | N | N/A | N/A | N/A | N/A |
| 27 | S2 | 2018/19 | Ill | Weaner | N | N | N | N | N | N/A | N/A | N/A | N/A |
| 28 | S2 | 2018/19 | Ill | Weaner | N | N | N | N | N | N/A | N/A | N/A | N/A |
| 29 | S2 | 2018/19 | Ill | Grower | N | N | N/A | N/A | N/A | N/A | N/A | N/A | N/A |
| 30 | S2 | 2018/19 | Ill | Weaner | N | N | N/A | N/A | N/A | N/A | N/A | N/A | N/A |
| 31 | S2 | 2018/19 | Ill | Weaner | N | N | N/A | N/A | N/A | N/A | N/A | N/A | N/A |
| 32 | S2 | 2018/19 | Ill | Weaner | N | N | N/A | N/A | N/A | N/A | N/A | N/A | N/A |
| 33 | S2 | 2018/19 | Ill | Weaner | N | N | N/A | N/A | N/A | N/A | N/A | N/A | N/A |
| 34 | S2 | 2016/17 | Ill | Weaner | N | N/A | N/A | N/A | N/A | N/A | N/A | N/A | N/A |
| 35 | S2 | 2016/17 | Ill | Weaner | N | N/A | N/A | N/A | N/A | N/A | N/A | N/A | N/A |
| 36 | S2 | 2016/17 | Ill | Weaner | N | N/A | N/A | N/A | N/A | N/A | N/A | N/A | N/A |
| 37 | S2 | 2016/17 | Ill | Weaner | N | N/A | N/A | N/A | N/A | N/A | N/A | N/A | N/A |
| 38 | S2 | 2016/17 | Ill | Weaner | N | N/A | N/A | N/A | N/A | N/A | N/A | N/A | N/A |
| 39 | S2 | 2016/17 | Ill | Weaner | N | N/A | N/A | N/A | N/A | N/A | N/A | N/A | N/A |
| 40 | S2 | 2016/17 | Ill | Weaner | N | N/A | N/A | N/A | N/A | N/A | N/A | N/A | N/A |
| 41 | S2 | 2016/17 | Ill | Weaner | N | N/A | N/A | N/A | N/A | N/A | N/A | N/A | N/A |
| 42 | S2 | 2016/17 | Ill | Weaner | N | N/A | N/A | N/A | N/A | N/A | N/A | N/A | N/A |
| 43 | S2 | 2016/17 | Ill | Weaner | N | N/A | N/A | N/A | N/A | N/A | N/A | N/A | N/A |
| 44 | S2 | 2016/17 | Ill | Weaner | N | N/A | N/A | N/A | N/A | N/A | N/A | N/A | N/A |
| 45 | S2 | 2016/17 | Ill | Weaner | N | N/A | N/A | N/A | N/A | N/A | N/A | N/A | N/A |
| 46 | S2 | 2016/17 | Ill | Weaner | N | N/A | N/A | N/A | N/A | N/A | N/A | N/A | N/A |
| 47 | S2 | 2016/17 | Ill | Weaner | + | N/A | N/A | N/A | N/A | N/A | N/A | N/A | N/A |
| 48 | S2 | 2016/17 | Ill | Weaner | + | N/A | N/A | N/A | N/A | N/A | N/A | N/A | N/A |
| **49** | **S3** | **2018/19** | **Ill** | **Weaner** | + | N | N | N | N | N | N | N | N |
| **50** | **S3** | **2018/19** | **Ill** | **Weaner** | **+** | **+** | **+** | **+** | **+** | **+** | **+** | **+** | **+** |
| **51** | **S3** | **2018/19** | **Ill** | **Weaner** | **N** | **+** | **+** | **N/A** | **+** | **N** | **N** | **N** | **N** |
| **52** | **S3** | **2018/19** | **Ill** | **Weaner** | **N** | **+** | **N** | **N** | **+** | **N** | **N** | **N** | **N/A** |
| **53** | **S3** | **2018/19** | **Ill** | **Weaner** | **N** | **+** | **N** | **+** | **N** | **N** | **N** | **N/A** | **N/A** |
| 54 | S3 | 2018/19 | Ill | Weaner | N | N | N/A | N/A | N/A | N/A | N/A | N/A | N/A |
| **55** | **S3** | **2018/19** | **Ill** | **Weaner** | **+** | **+** | **N** | **N** | **N** | **N/A** | **N/A** | **N/A** | **N/A** |
| 56 | S3 | 2018/19 | Ill | Weaner | N | N | N | N | N | N/A | N/A | N/A | N/A |
| **57** | **S3** | **2018/19** | **Ill** | **Weaner** | **+** | **+** | **+** | **+** | **N** | **N/A** | **N/A** | **N/A** | **N/A** |
| 58 | S3 | 2018/19 | Ill | Weaner | N | N | N/A | N/A | N/A | N/A | N/A | N/A | N/A |
| 59 | S4 | 2018/19 | Ill | Weaner | N | N | N/A | N/A | N/A | N/A | N/A | N/A | N/A |
| 60 | S4 | 2018/19 | Ill | Grower | N | N | N/A | N/A | N/A | N/A | N/A | N/A | N/A |
| 61 | S4 | 2018/19 | Ill | Grower | N | N | N/A | N/A | N/A | N/A | N/A | N/A | N/A |
| 62 | S4 | 2018/19 | Ill | Weaner | N | N | N/A | N/A | N/A | N/A | N/A | N/A | N/A |
| 63 | S4 | 2018/19 | Ill | Weaner | N | N | N/A | N/A | N/A | N/A | N/A | N/A | N/A |
| 64 | S4 | 2018/19 | Ill | Weaner | N | N | N/A | N/A | N/A | N/A | N/A | N/A | N/A |
| 65 | S4 | 2018/19 | Ill | Weaner | N | N | N/A | N/A | N/A | N/A | N/A | N/A | N/A |
| 66 | S5 | 2018/19 | Ill | Fetus | N/A | N/A | N/A | N/A | N/A | N | N/A | N/A | N/A |
| 67 | S5 | 2018/19 | Ill | Weaner | N | N/A | N/A | N/A | N/A | N/A | N/A | N/A | N/A |
| 68 | S5 | 2018/19 | Ill | Weaner | N | N/A | N/A | N/A | N/A | N/A | N/A | N/A | N/A |
| 69 | S5 | 2018/19 | Ill | Grower | N | N/A | N/A | N/A | N/A | N/A | N/A | N/A | N/A |
| 70 | S5 | 2018/19 | Ill | Piglet | N | N/A | N/A | N/A | N/A | N/A | N/A | N/A | N/A |
| 71 | S5 | 2018/19 | Ill | Grower | N/A | N | N/A | N/A | N/A | N/A | N/A | N/A | N/A |
| 72 | S5 | 2018/19 | Ill | Grower | N/A | N | N/A | N/A | N/A | N/A | N/A | N/A | N/A |
| 73 | S6 | 2018/19 | Ill | Weaner | N | N/A | N/A | N/A | N/A | N/A | N/A | N/A | N/A |
| 74 | S7 | 2016/17 | Ill | Weaner | + | N/A | N/A | N/A | N/A | N/A | N/A | N/A | N/A |
| 75 | S8 | 2018/19 | Ill | Foetus | N | N/A | N/A | N/A | N/A | N | N/A | N/A | N/A |
| 76 | S9 | 2018/19 | Ill | Foetus | N/A | N/A | N/A | N/A | N | N | N/A | N/A | N/A |
| 77 | S9 | 2018/19 | Ill | Foetus | N/A | N/A | N/A | N/A | N | N | N/A | N/A | N/A |
| 78 | S9 | 2018/19 | Ill | Foetus | N/A | N/A | N/A | N/A | N | N | N/A | N/A | N/A |
| 79 | S9 | 2018/19 | Ill | Foetus | N | N | N/A | N/A | N/A | N | N/A | N/A | N/A |
| 80 | K1 | 2016/17 | Ill | Weaner | + | N/A | N/A | N/A | N/A | N/A | N/A | N/A | N/A |
| 81 | K1 | 2016/17 | Ill | Weaner | N | N/A | N/A | N/A | N/A | N/A | N/A | N/A | N/A |
| 82 | K1 | 2016/17 | Ill | Weaner | N | N/A | N/A | N/A | N/A | N/A | N/A | N/A | N/A |
| 83 | K1 | 2016/17 | Ill | Weaner | N | N/A | N/A | N/A | N/A | N/A | N/A | N/A | N/A |
| 84 | K2 | 2018/19 | Ill | Piglet | N/A | N/A | N/A | N/A | N/A | N | N/A | N/A | N/A |
| 85 | K2 | 2018/19 | Ill | Piglet | N | N | N/A | N/A | N/A | N | N/A | N/A | N/A |
| 86 | K2 | 2018/19 | Ill | Piglet | N/A | N/A | N/A | N/A | N/A | N | N/A | N/A | N/A |
| 87 | K3 | 2018/19 | Ill | Grower | N | N/A | N | N | N/A | N/A | N/A | N/A | N/A |
| **88** | **K3** | **2018/19** | **Ill** | **Grower** | **+** | **N/A** | **+** | **N/A** | **N/A** | **N/A** | **N** | **N** | **N/A** |
| 89 | K3 | 2018/19 | Ill | Weaner | N | N/A | N/A | N/A | N/A | N/A | N/A | N/A | N/A |
| 90 | K4 | 2018/19 | Ill | Weaner | N | N | N/A | N/A | N/A | N/A | N/A | N/A | N/A |
| 91 | K4 | 2018/19 | Ill | Weaner | N | N | N/A | N/A | N/A | N/A | N/A | N/A | N/A |
| **92** | **K5** | **2018/19** | **Ill** | **Grower** | **+** | **N/A** | **N** | **N** | **N/A** | **N/A** | **N** | **N/A** | **N/A** |
| **93** | **K5** | **2018/19** | **Ill** | **Grower** | **+** | **N/A** | **N** | **N/A** | **N/A** | **N/A** | **N** | **N** | **N/A** |
| 94 | M1 | 2016/17 | Ill | Weaner | N | N/A | N/A | N/A | N/A | N/A | N/A | N/A | N/A |
| 95 | M1 | 2016/17 | Ill | Weaner | N | N/A | N/A | N/A | N/A | N/A | N/A | N/A | N/A |
| 96 | M1 | 2016/17 | Ill | Weaner | N | N/A | N/A | N/A | N/A | N/A | N/A | N/A | N/A |
| 97 | M1 | 2016/17 | Ill | Weaner | N | N/A | N/A | N/A | N/A | N/A | N/A | N/A | N/A |
| 98 | M1 | 2016/17 | Ill | Weaner | N | N/A | N/A | N/A | N/A | N/A | N/A | N/A | N/A |
| 99 | M2 | 2018/19 | Ill | Weaner | N | N/A | N/A | N/A | N/A | N/A | N/A | N/A | N/A |
| 100 | M2 | 2018/19 | Ill | Weaner | N | N/A | N/A | N/A | N/A | N/A | N/A | N/A | N/A |
| 101 | M3 | 2018/19 | Ill | Grower | + | N/A | N/A | N/A | N/A | N/A | N/A | N/A | N/A |
| 102 | M3 | 2018/19 | Ill | Grower | + | N/A | N/A | N/A | N/A | N/A | N/A | N/A | N/A |
| 103 | J1 | 2016/17 | Ill | Weaner | + | N/A | N/A | N/A | N/A | N/A | N/A | N/A | N/A |
| 104 | J1 | 2016/17 | Ill | Weaner | N | N/A | N/A | N/A | N/A | N/A | N/A | N/A | N/A |
| 105 | J1 | 2016/17 | Ill | Weaner | N | N/A | N/A | N/A | N/A | N/A | N/A | N/A | N/A |
| 106 | J2 | 2018/19 | Ill | Piglet | N | N | N/A | N/A | N/A | N | N/A | N/A | N/A |
| 107 | J3 | 2018/19 | Ill | Grower | N | N | N | N | N | N/A | N/A | N/A | N/A |
| 108 | P1 | 2016/17 | Ill | Weaner | N | N/A | N/A | N/A | N/A | N/A | N/A | N/A | N/A |
| 109 | P1 | 2016/17 | Ill | Weaner | N | N/A | N/A | N/A | N/A | N/A | N/A | N/A | N/A |
| 110 | P1 | 2016/17 | Ill | Weaner | N | N/A | N/A | N/A | N/A | N/A | N/A | N/A | N/A |
| 111 | P1 | 2016/17 | Ill | Weaner | N | N/A | N/A | N/A | N/A | N/A | N/A | N/A | N/A |
| 112 | P1 | 2016/17 | Ill | Weaner | N | N/A | N/A | N/A | N/A | N/A | N/A | N/A | N/A |
| 113 | P1 | 2016/17 | Ill | Weaner | N | N/A | N/A | N/A | N/A | N/A | N/A | N/A | N/A |
| 114 | P1 | 2016/17 | Ill | Weaner | N | N/A | N/A | N/A | N/A | N/A | N/A | N/A | N/A |
| 115 | P2 | 2018/19 | Ill | Grower | N | N | N/A | N/A | N/A | N/A | N/A | N/A | N/A |
| 116 | P2 | 2018/19 | Ill | Grower | N | N | N/A | N/A | N/A | N/A | N/A | N/A | N/A |
| 117 | P2 | 2018/19 | Ill | Grower | N | N | N/A | N/A | N/A | N/A | N/A | N/A | N/A |
| 118 | P3 | 2018/19 | Ill | Grower | N | N | N/A | N/A | N/A | N/A | N/A | N/A | N/A |
| 119 | P4 | 2018/19 | Ill | Grower | N | N | N/A | N/A | N/A | N/A | N/A | N/A | N/A |
| 120 | P4 | 2018/19 | Ill | Weaner | N | N | N/A | N/A | N/A | N/A | N/A | N/A | N/A |
| **121** | **P4** | **2018/19** | **Ill** | **Grower** | **N** | **+** | **+** | **+** | **+** | **N/A** | **+** | **N/A** | **N/A** |
| 122 | P4 | 2018/19 | Ill | Weaner | N | N | N/A | N/A | N/A | N/A | N/A | N/A | N/A |
| 123 | P4 | 2018/19 | Ill | Weaner | N | N | N/A | N/A | N/A | N/A | N/A | N/A | N/A |
| 124 | K3 | 2018/19 | Healthy | Finisher | N | N/A | N/A | N/A | N/A | N/A | N/A | N/A | N/A |
| 125 | K3 | 2018/19 | Healthy | Finisher | N | N/A | N/A | N/A | N/A | N/A | N/A | N/A | N/A |
| 126 | K3 | 2018/19 | Healthy | Finisher | N | N/A | N/A | N/A | N/A | N/A | N/A | N/A | N/A |
| 127 | K3 | 2018/19 | Healthy | Finisher | N | N/A | N/A | N/A | N/A | N/A | N/A | N/A | N/A |
| 128 | K3 | 2018/19 | Healthy | Finisher | N | N/A | N/A | N/A | N/A | N/A | N/A | N/A | N/A |
| 129 | K3 | 2018/19 | Healthy | Finisher | N | N/A | N/A | N/A | N/A | N/A | N/A | N/A | N/A |
| 130 | K3 | 2018/19 | Healthy | Finisher | N | N/A | N/A | N/A | N/A | N/A | N/A | N/A | N/A |
| 131 | K3 | 2018/19 | Healthy | Finisher | N | N/A | N/A | N/A | N/A | N/A | N/A | N/A | N/A |
| 132 | K3 | 2018/19 | Healthy | Finisher | N | N/A | N/A | N/A | N/A | N/A | N/A | N/A | N/A |
| 133 | K3 | 2018/19 | Healthy | Finisher | N | N/A | N/A | N/A | N/A | N/A | N/A | N/A | N/A |
| 134 | K3 | 2018/19 | Healthy | Finisher | N | N/A | N/A | N/A | N/A | N/A | N/A | N/A | N/A |
| 135 | K3 | 2018/19 | Healthy | Finisher | N | N/A | N/A | N/A | N/A | N/A | N/A | N/A | N/A |
| 136 | K3 | 2018/19 | Healthy | Finisher | N | N/A | N/A | N/A | N/A | N/A | N/A | N/A | N/A |
| 137 | K3 | 2018/19 | Healthy | Finisher | N | N/A | N/A | N/A | N/A | N/A | N/A | N/A | N/A |
| 138 | K3 | 2018/19 | Healthy | Finisher | N | N/A | N/A | N/A | N/A | N/A | N/A | N/A | N/A |
| 139 | K3 | 2018/19 | Healthy | Finisher | N | N/A | N/A | N/A | N/A | N/A | N/A | N/A | N/A |
| 140 | S2 | 2018/19 | Healthy | Finisher | N | N/A | N/A | N/A | N/A | N/A | N/A | N/A | N/A |
| 141 | S2 | 2018/19 | Healthy | Finisher | N | N/A | N/A | N/A | N/A | N/A | N/A | N/A | N/A |
