# Supplementary material for: First molecular detection and complete sequence analysis of porcine circovirus type 3 (PCV3) in Peninsular Malaysia
Source: PLoS One. 2020 Jul 24;15(7):e0235832. doi: 10.1371/journal.pone.0235832 (PMC7380639; doi:10.1371/journal.pone.0235832)
Supplement: S3 Table — Statistically significant values are highlighted with grey boxes and boldface. (DOC) [file pone.0235832.s003.doc]

Supplementary Table 3. Detailed statistical calculation for Chi-square and Fisher's exact tests evaluating association between PCV3 molecular detection status and age group, health status, farm standing sow population, distance from neighbouring farms, and across different organs. Statistically significant values are highlighted with grey boxes and boldface.	

i)	Crosstabulation of PCV3 molecular detection status and age group
 
Age Group
Total


Finisher & Sow
Grower
Piglet
Fetus
Weaner


PCV3
 
NEG
 
Count
18
18
5
6
70
117


Expected Count
14.9
15.8
4.1
5
77.2
117


% within PCV3
15.40%
15.40%
4.30%
5.10%
59.80%
100.00%


Adjusted Residual
2.1
1.5
1
1.1
-3.4
 


 Post Hoc 
0.036
0.133
0.317
0.271
0.0007
 


POS
 
Count
0
1
0
0
23
24


Expected Count
3.1
3.2
0.9
1
15.8
24


% within PCV3
0.00%
4.20%
0.00%
0.00%
95.80%
100.00%


Adjusted Residual
-2.1
-1.5
-1
-1.1
3.4
 


 Post Hoc
0.036
0.133
0.317
0.271
0.0007
 

a.	Adjusted p < 0.005	
Value
df
Asymptotic Significance (2-sided)
p-value

Pearson Chi-Square
11.723a
4
0.020
0.024

Likelihood Ratio
16.778
4
0.002
0.002

Fisher's Exact Test
10.243


0.022

N of Valid Cases
141


a.	6 cells (60.0%) have expected count less than 5. The minimum expected count is 0.85.
b.	Statistical significance was set at p < 0.05.	

ii)	Crosstabulation of PCV3 molecular detection status and health status 

Health Status
Total


Clinically healthy
Clinically ill


PCV3
 
NEG
 
Count
18
99
117


Expected Count
14.9
102.1
117.0


% within PCV3
15.4%
84.6%
100.0%


POS
 
Count
0
24
24


Expected Count
3.1
20.9
24.0


% within PCV3
0.0%
100.0%
100.0%

	
Value
df
Asymptotic Significance (2-sided)
p-value

Pearson Chi-Square
4.233a
1
0.040


Continuity Correctionb
2.964
1
0.085


Likelihood Ratio
7.238
1
0.007


Fisher's Exact Test


0.043

N of Valid Cases
141


a.	1 cell (25.0%) have expected count less than 5. The minimum expected 
count is 3.06
b.	Computed only for a 2x2 table.
c.	Statistical significance was set at p < 0.05	

iii)	Crosstabulation of PCV3 molecular detection status and farm standing sow population

SowSPP
Total


< 800 heads
> 800 heads


PCV3
NEG
Count
58
59
117


Expected Count
54.8
62.2
117.0


% within PCV3
49.6%
50.4%
100.0%


POS
Count
8
16
24


Expected Count
11.2
12.8
24.0


% within PCV3
33.3%
66.7%
100.0%

	
Value
df
Asymptotic Significance (2-sided)
p-value

Pearson Chi-Square
2.109a
1
0.146
0.180

Continuity Correctionb
1.508
1
0.220


Likelihood Ratio
2.152
1
0.142
0.180

Fisher's Exact Test


0.180

N of Valid Cases
141


a.	0 cells (0.0%) have expected count less than 5. The minimum 
expected count is 11.23.					
b.	Statistical significance was set at p < 0.05.	

iv)	Crosstabulation of PCV3 molecular detection status and distance from neighbouring farms

Radius
Total


<1 km
>10 km
1 – 10 km


PCV3
NEG
Count
88
20
9
117


Expected Count
88.8
20.7
7.5
117.0


% within PCV3
75.2%
17.1%
7.7%
100.0%


POS
Count
19
5
0
24


Expected Count
18.2
4.3
1.5
24.0


% within PCV3
79.2%
20.8%
0.0%
100.0%

	
Value
df
Asymptotic Significance (2-sided)
p-value

Pearson Chi-Square
2.044a
2
0.360
0.449

Likelihood Ratio
3.549
2
0.170
0.287

Fisher's Exact Test
1.667


0.512

N of Valid Cases
141


a.	2 cells (33.3%) have expected count less than 5. The minimum expected count is 1.53
b.	Statistical significance was set at p < 0.05.	


v)	
Crosstabulation of PCV3 molecular detection status across different organs

Tissues
Total


Brain
Heart
ILN
Kidney
Liver
Lung
MLN
Spleen
Tonsil


PCV3
 
NEG
 
Count
3
5
2
6
7
4
8
7
5
47


Expected Count
2.1
3.1
5.7
5.7
4.1
7.2
6.2
7.2
5.7
39


% within PCV3
6.4%
10.6%
4.3%
12.8%
14.9%
8.5%
17.0%
14.9%
10.6%
100.0%


Residual
1.0
1.6
-2.4
.2
2.1
-1.9
1.1
-0.1
-0.4


Post Hoc
0.339
0.108
0.018
0.838
0.034
0.060
0.264
0.893
0.661
 


POS
 
Count
1
1
9
5
1
10
4
7
6
43


Expected Count
1.9
2.9
5.3
5.3
3.9
6.8
5.8
6.8
5.3
43.0


% within PCV3
2.3%
2.3%
20.5%
11.4%
2.3%
22.7%
9.1%
15.9%
13.6%
100.0%


Residual
-1.0
-1.6
2.4
-0.2
-2.1
1.9
-1.1
0.1
0.4


PostHoc
0.339
0.108
0.018
0.838
0.034
0.060
0.264
0.893
0.661
 

	
Value
df
Asymptotic Significance (2-sided)
p-value

Pearson Chi-Square
16.627a
8
0.034
0.029

Likelihood Ratio
17.937
8
0.022
0.037

Fisher's Exact Test
16.199
 
 
0.032

N of Valid Cases
82


a.	9 cells (50.0%) have expected count less than 5. The minimum expected count is 1.43
b.	Statistical significance was set at p < 0.05.	
a.	Adjusted p-value was determined to be < 0.003
               
