# Supplementary material for: First molecular detection and complete sequence analysis of porcine circovirus type 3 (PCV3) in Peninsular Malaysia
Source: PLoS One. 2020 Jul 24;15(7):e0235832. doi: 10.1371/journal.pone.0235832 (PMC7380639; doi:10.1371/journal.pone.0235832)
Supplement: S4 Table — Twelve Malaysian PCV3 strains and 30 PCV3 GenBank reference strains were compared. The analysis was run using Pairwise Distance method, p-distance method evaluated with 1000 bootstrap replicates. P-distance values of ≥ 0.020 are indicated in grey boxes. (DOCX) [file pone.0235832.s004.docx]

**Supplementary Table 4. Pairwise distance analysis of complete genomes of PCV3, shown as p-distance values.**

Twelve Malaysian PCV3 strains and 30 PCV3 GenBank reference strains were compared. Malaysian PCV3 strains are highlighted in black textboxes. The analysis was run using Pairwise Distance method, p-distance method evaluated with 1000 bootstrap replicates. P-distance values and their respective standard error estimates are tabulated in corresponding diagonal positions. P-distance values of ≥ 0.020 are indicated in grey boxes.
