# Supplementary material for: First molecular detection and complete sequence analysis of porcine circovirus type 3 (PCV3) in Peninsular Malaysia
Source: PLoS One. 2020 Jul 24;15(7):e0235832. doi: 10.1371/journal.pone.0235832 (PMC7380639; doi:10.1371/journal.pone.0235832)
Supplement: S7 Table — Fifteen Malaysian PCV3 strains and 30 PCV3 GenBank reference strains were analysed for p-distance values as described in S6 Table. P-distance values are represented as percentage nt identities here in S7 Table. Percentage nt identities of ≤ 97.36% are indicated in grey boxes. (DOCX) [file pone.0235832.s007.docx]

**Supplementary Table 7. Pairwise distance analysis of *cap* gene sequences of PCV3, shown as percentage nucleotide identities.**

Fifteen Malaysian PCV3 strains and 30 PCV3 GenBank reference strains were analysed for p-distance values as described in Supplementary Table 6. Malaysian PCV3 strains are highlighted in black textboxes. The resulting p-distance values are represented as percentage nucleotide identities here in Supplementary Table 7. Percentage nt identities of ≤ 97.36% are indicated in grey boxes.
